# The Peripheral Blood Transcriptome Is Correlated With PET Measures of Lung Inflammation During Successful Tuberculosis Treatment

**DOI:** 10.3389/fimmu.2020.596173

**Published:** 2021-02-10

**Authors:** Trust Odia, Stephanus T. Malherbe, Stuart Meier, Elizna Maasdorp, Léanie Kleynhans, Nelita du Plessis, Andre G. Loxton, Daniel E. Zak, Ethan Thompson, Fergal J. Duffy, Helena Kuivaniemi, Katharina Ronacher, Jill Winter, Gerhard Walzl, Gerard Tromp, the Catalysis TB-Biomarker Consortium, André G. Loxton

**Affiliations:** ^1^ Division of Molecular Biology and Human Genetics, Department of Biomedical Sciences, Stellenbosch University, Cape Town, South Africa; ^2^ DSI–NRF Centre of Excellence for Biomedical Tuberculosis Research, Stellenbosch University, Cape Town, South Africa; ^3^ South African Medical Research Council Centre for Tuberculosis Research, Stellenbosch University, Cape Town, South Africa; ^4^ Bioinformatics Unit, South African Tuberculosis Bioinformatics Initiative, Stellenbosch University, Cape Town, South Africa; ^5^ Centre for Bioinformatics and Computational Biology, Stellenbosch University, Stellenbosch, South Africa; ^6^ Center for Infectious Disease Research, Seattle, WA, United States; ^7^ Seattle Children’s Research Institute, Center for Global Infectious Disease Research, Seattle, WA, United States; ^8^ Translational Research Institute, Mater Research Institute - The University of Queensland, Brisbane, QLD, Australia; ^9^ Catalysis Foundation for Health, San Ramon, CA, United States

**Keywords:** gene expression, [^18^F]FDG PET-CT, RNA-sequencing, mixed-effect models, pathway analysis, transcription factor binding site, tuberculosis, treatment response

## Abstract

Pulmonary tuberculosis (PTB) is characterized by lung granulomas, inflammation and tissue destruction. Here we used within-subject peripheral blood gene expression over time to correlate with the within-subject lung metabolic activity, as measured by positron emission tomography (PET) to identify biological processes and pathways underlying overall resolution of lung inflammation. We used next-generation RNA sequencing and [^18^F]FDG PET-CT data, collected at diagnosis, week 4, and week 24, from 75 successfully cured PTB patients, with the [^18^F]FDG activity as a surrogate for lung inflammation. Our linear mixed-effects models required that for each individual the slope of the line of [^18^F]FDG data in the outcome and the slope of the peripheral blood transcript expression data correlate, i.e., the slopes of the outcome and explanatory variables had to be similar. Of 10,295 genes that changed as a function of time, we identified 639 genes whose expression profiles correlated with decreasing [^18^F]FDG uptake levels in the lungs. Gene enrichment over-representation analysis revealed that numerous biological processes were significantly enriched in the 639 genes, including several well known in TB transcriptomics such as platelet degranulation and response to interferon gamma, thus validating our novel approach. Others not previously associated with TB pathobiology included smooth muscle contraction, a set of pathways related to mitochondrial function and cell death, as well as a set of pathways connecting transcription, translation and vesicle formation. We observed up-regulation in genes associated with B cells, and down-regulation in genes associated with platelet activation. We found 254 transcription factor binding sites to be enriched among the 639 gene promoters. In conclusion, we demonstrated that of the 10,295 gene expression changes in peripheral blood, only a subset of 639 genes correlated with inflammation in the lungs, and the enriched pathways provide a description of the biology of resolution of lung inflammation as detectable in peripheral blood. Surprisingly, resolution of PTB inflammation is positively correlated with smooth muscle contraction and, extending our previous observation on mitochondrial genes, shows the presence of mitochondrial stress. We focused on pathway analysis which can enable therapeutic target discovery and potential modulation of the host response to TB.

## Introduction

Tuberculosis (TB) is among the leading causes of mortality due to infectious diseases worldwide, with 1.5 million deaths recorded in 2018 (1). TB is caused by *Mycobacterium tuberculosis* and transmitted *via* inhalation of air droplets expelled by a person with active TB. The primary site of *M. tuberculosis* infection is the lung, which leads to pulmonary TB (PTB).

Lung inflammation seen in PTB is a result of the effector functions of host immune cells at the site of infection. In the absence of outright eradication of *M. tuberculosis* at the site, an accumulation of immune cells around invading bacteria leads to granuloma formation in an attempt to control infection. During disease progression, persistent lung inflammation results in the coalescence of granulomas into larger lung lesions with concomitant necrosis and formation of cavities (2, 3). Cavities can be detected using radiological methods such as chest X-ray and computed tomography (CT) (4, 5).

Positron emission tomography-computed tomography (PET-CT) is a medical imaging method, in which PET provides functional and CT structural information (6–10). ^18^F-labeled fluorodeoxyglucose (FDG) is a common PET tracer, and its accumulation in tissues of the body indicates enhanced glucose metabolism (11–17), which, in inflammatory diseases, can be used as a surrogate for the extent of inflammation (18). PET-CT is, however, a very expensive procedure (19) that is not readily available in resource-limited settings with high TB incidence and exposes subjects to radiation (20, 21) while not providing any information about underlying pathophysiological changes.

A number of prior studies have identified gene expression signatures from whole blood which reflect changes in transcript levels in response to TB as well as response to treatment (22–30). Some studies aimed at developing blood transcriptomic signatures for diagnosis of active (31, 32) or incipient (28, 33) TB showed that the signatures correlated with [^18^F]FDG uptake levels. Most of these studies focused primarily on biomarker discovery by analyzing group differences between timepoints in peripheral blood (24, 25, 28, 31). In contrast to previous studies (31, 33, 34), we focused on elucidation of biology by constraining the transcriptional changes to resolution of lung inflammation, i.e., correlation with the PET metrics, within subject over time. Previous studies have suggested correlation between peripheral blood transcriptional changes and the resolution of lung inflammation (28, 31–33, 35). Here we used a linear mixed models approach to model the response of each subject separately using the [^18^F]FDG decrease over time as an outcome variable and requiring that the slope of the gene expression over time as an explanatory variable correspond with that of the [^18^F]FDG. That is, there had to be within-subject correlation between outcome and explanatory variable, and then we modeled to overall groupwise trends. This modeling constrains the gene response to be tightly correlated with the [^18^F]FDG metric and therefore with resolution of lung inflammation. Gene expression and PET-CT measurements were analyzed from 75 patients who were considered cured based on microbiological tests during the course of successful treatment (16). Using our models, we were able to account for the inherent intra- and inter-subject correlation and accommodate missing data as well as the hierarchical data structure. Linear mixed-effect models are statistically more powerful than merely modelling the means of groups, as is done in more conventional statistical analyses (36). In contrast to the 10,295 genes that changed as a function of time, our method identified 639 genes whose expression levels were consistent with decreasing [^18^F]FDG activity in the lung, during PTB treatment. These genes could reveal more about the inflammation-related biological processes involved in the lung disease than models that do not account for within subject variation and do not constrain the transcriptional changes to the changes in PET metrics. We also performed cellular deconvolution with the transcriptomics data to determine the influence of cell type on the modeling, and found that variations in cell proportions change the genes and pathways identified as correlated with PET metrics.

## Materials and Methods

### Study Design

**Figure 1:**
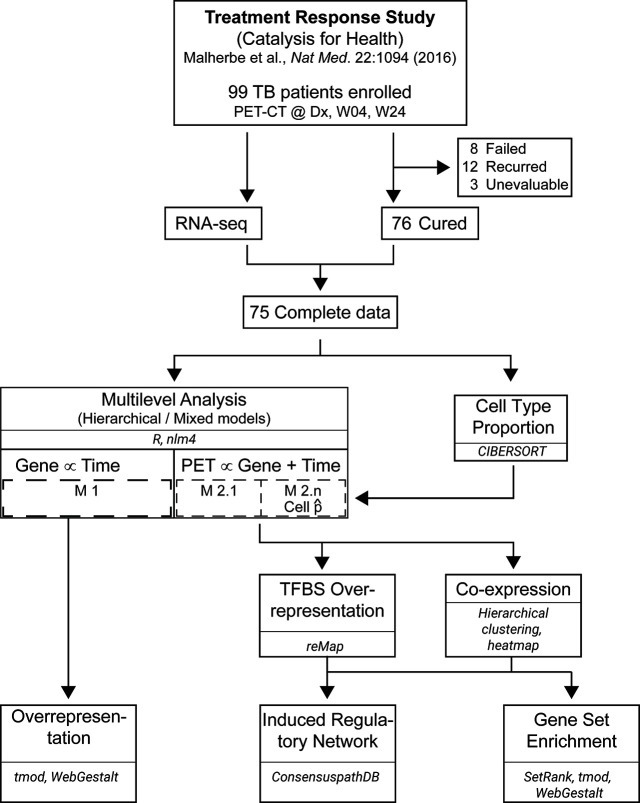
Workflow for data analysis. RNA-seq data from 75 PTB patients at Dx, Week 4 (W04) and Week 24 (W24) was merged with [^18^F]FDG PET-CT data from 75 patients at the same time points. Linear mixed-effect models with varying intercepts, and varying slopes were built with lme4 in R. Whole blood deconvolution of RNA-seq data was performed with CIBERSORT, and the proportions of naïve B cells, CD8^+^ αβ T cells, CD14^+^ monocytes and neutrophils were used as a covariable in the models. Transcription factor binding sites (TFBS) over-presentation and co-expression analyses were performed on the results from Models 2.1 to 2.5. The results from co-expression analysis were used to construct an induced gene regulatory network and perform gene set enrichment. Overrepresentation analysis was also performed on the genes from Model 1.

We analyzed [^18^F]FDG PET-CT scan metrics and whole blood RNA-sequencing (RNA-seq) data available from study subjects (**Figures 1** and **S1**, and **Table 1**). In previously published studies (16, 28, 37), 99 patients were followed up during TB treatment from diagnosis (Dx), week 1 (W01), week 4 (W04), to week 24 (W24). Approval was obtained from the Health Research Ethics Committee (HREC) of Stellenbosch University (registration number N10/01/013), to recruit patients and collect specimens. For the current study to re-analyze the PET-CT and mRNA expression data, we received a separate HREC approval (X18/09/029).

**Table 1:** Summary of available data.

RNA-seq						PET-CT					RNA-seq and PET-CT				
Dx	W01	W04	W24	N		Dx	W04	W24	N		Dx	W04	W24	N
			P	1		P	P	P	76				P	1^a^
		P	P	2								P	P	5
	P	P	P	3							P		P	5
P			P	1							P	P		1
P		P	P	1							P	P	P	64
P	P		P	4										
P	P	P		1										
P	P	P	P	63										
Total				76					76					76

Dx, diagnosis; W01, week 1; W04, week 4; W24, week 24; N, number of subjects; P, data available.

a Subject removed from analysis, since RNA-seq data were available for only one-time point.

PTB patients for the original study were recruited from the local communities or at Tygerberg Academic Hospital in Cape Town, South Africa. They were 16 – 70 years old, had no other lung disease, were non-diabetic, and were HIV-negative at diagnosis. They had also not been on any steroid medication in the past 6 months, were not pregnant and did not have cancer (for a detailed list of inclusion and exclusion criteria, see **Supplementary Methods**). The study patients received treatment as prescribed by the South African National Tuberculosis Programme, based on WHO guidelines. This consisted of 6 months of combination therapy: 2 months of intensive phase with rifampicin, isoniazid, pyrazinamide and ethambutol daily, followed by 4 months of continuation phase with rifampicin and isoniazid daily (for details, see **Supplementary Methods**) (1, 16). All subjects were diagnosed with active TB by microbiological sputum culture examination at Dx. After 2 years of follow-up, 76 patients were designated “cured”; 8 “failed treatment”; 12 “recurrent TB”; and 3 “unevaluable” by microbiological sputum culture at the end of treatment (W24).

RNA-seq was performed on specimens from time points: Dx, W04 and W24. We used 75 cured subjects out of the 76, who had [^18^F]FDG PET-CT scans and RNA-seq data available at a minimum of 2 time points (**Table 1**). We chose to use all available data, i.e., perform an incomplete-case data analysis since 85% of the cases were complete and would stabilize the estimates against wild swings from the incomplete cases. Additionally, another 6% of the data consisted of start and end timepoints which should result in less divergence of the slope estimates than cases missing either the first or the last timepoint. There is extensive literature that indicates that performing a complete-case analysis will lead to greater bias than performing an incomplete-case analysis (i.e., all available data, cases with complete data plus those with missing data) (38–50). One approach to incomplete data is imputation. In this instance imputation was not possible. Fortunately, linear mixed models as implemented in generalized linear mixed models are robust against missingness even under less restrictive assumptions (i.e., missing at random as opposed to missing completely at random), thus using incomplete-case data is plausible for the current analyses (48, 51).

We downloaded log_2_ transformed RNA-seq read counts from Gene Expression Omnibus (GSE89403) (28). Read pairs were previously aligned and mapped to human genome hg19 with **STAR** version 2.3.1d (52), and **htseq** version 0.6.0 (53) was used for counting the overlaps of reads with genes. Read counts were normalized with the **cpm** function in **edgeR** package (54) in **R** (55), and transformed to log_2_ values, hence no further pre-processing or quality control steps were performed on the data in this study. Reads were mapped to transcripts identified by Ensembl IDs and were mapped to Entrez Gene IDs using **biomaRt** (56).

### PET-CT Metrics

We had PET-CT scan data at time points Dx, W04 and W24 for 75 “Cured” subjects, whose RNA-seq data were available (**Table 1**). In previous studies (57, 58), mean standardized lesion activity (MSLA) was one of the quantified PET metrics. MSLA is a normalized mean intensity of [^18^F]FDG uptake in the area of the lung above a background threshold, computed as Z-score (58). It describes mean lesion intensity in a specified area of the lung. MSLA decreased nearly linearly over time, when time was treated as an ordinal (ordinal time in weeks corresponds approximately to the cube root of weeks). MSLA was also characterized by large variance (**Figure S2**), hence we included it in our models as an outcome variable. MSLA is a PET metric, previously used to calculate [^18^F]FDG lung lesion activity, and was reported to correlate with changes in lung inflammation (16, 57). It should be noted that few subjects had MSLA of 0 at the end of treatment, and that most (67 of 75) of the PET-CT scans were considered not resolved at the end of treatment. For statistical modeling, [^18^F]FDG PET-CT scan data were merged with whole blood RNA-seq data, using **R** version 3.6.1 (55).

### Estimation of Cell Proportion by Deconvolution

To estimate the proportions of lymphocyte types in the peripheral blood samples, we performed deconvolution using the gene expression counts and **CIBERSORT** (59) and the **immunoStates** expression matrix (60). The **CIBERSORT** deconvolution uses linear support vector regression together with an expression matrix of genes that are differential for one or more cell types. The immunoStates matrix comprises expression values for 317 genes and 20 cell types. We obtained estimates of cell proportions for all 20 cell types for each subject and timepoint for which data were available. For the cell types with non-trivial proportions and variance we performed a repeated measures ANOVA using **lme4** (61) in **R** (55) to determine if there was evidence for change in proportion over time.

### Statistical Modeling

We fit a linear mixed-effects, multi-level model (61, 62) that accounts for intra- and inter-subject variation. Mathematically the model is written as:

(1) $$y_{i}=\alpha_{ij}+\beta x_{i}+\epsilon_{i},for\;i=1,\ldots ,n$$

where *i* represents multiple values of the variable, *y_i_* is the outcome variable for the *i*-th individual at level one, in *j*-th group at level two, α*_ij_* is the varying-intercept, *x_i_* is level one predictor variable, *β* is the slope, and ϵ*_i_* is the level one random error. β represents the rate of change in *y* per unit in *x*. The predictor variables are the independent variables.

We fit a varying-intercept, and varying slope model; treating the subjects and gene expression levels, as random effects; and time, as a fixed effect (**Figure S1B**).

The models were formulated as follows:

Model 1, (2) $$G_{i}=T_{ij}+\left(1+T_{ij}|S_{j}\right)$$

where G is Gene, T is Time, S is Subject

Model 2, (3) $$M_{ij}=G_{ij}+T_{ij}+C_{ij}+\left(1+T_{ij}|S_{j}\right)$$

where M is MSLA, G is Gene, T is Time, S is Subject, C is Cell-type

In the equations, *i* is the subscript for time point (Dx, W04, W24) as an ordinal value; and *j* is the subscript for subject (n=75). Model 1 was used to extract the slope of each gene in each subject, while Model 2 constrains the expression levels of each gene to correlate with MSLA levels, over time, in each subject (**Figure S1**). In Model 2, we identified genes with significant model fit, using Satterthwaite’s method (63, 64), and Type III ANOVA F statistic test, implemented in **Anova** function in the **car** package (65) in **R** version 3.6.1 (55, 66, 67). We corrected for multiple testing using false discovery rate (FDR) (68), with the **p.adjust** function in **R**, and FDR < 0.05 was considered significant. The linear mixed-effects model imposes strict constraints on the data (RNA-seq, PET), such that the expression levels of the genes are correlated with PET levels, over time. Traditional differential gene expression analysis does not account for intra- and inter-subject variation.

Adjusting for cell type proportion entails adding variables to the model and incurs a penalty in terms of the degrees of freedom. Increasing the model complexity can result in false negatives due to this penalty. Correction for a single cell type alone leaves the result poorly interpretable, since significance can be driven by changes in cell proportion in any of the remaining cell types. We therefore selected a set of 4 cell types, i.e., naïve B cells, CD8^+^ αβ T cells, CD14^+^ monocytes and neutrophils and modeled these using a leave-one-out approach.

We generated results for 5 different analyses based on Model 2:

2.1) Base, a model without correction for cell type (*C_ij_* omitted)
2.2) B cell, a model correcting for CD8^+^ αβ T cells, CD14^+^ monocytes, and neutrophils
2.3) CD8 T cell, a model correcting for naïve B cells, CD14^+^ monocytes, and neutrophils
2.4) Monocyte, a model correcting for naïve B cells, CD8^+^ αβ T cells, and neutrophils
2.5) Neutrophil, a model correcting for naïve B cells, CD8^+^ αβ T cells, and CD14^+^ monocytes.

### Gene Set Enrichment Analysis

We performed three different gene set enrichment analyses, using the following tools: (a) gene set enrichment analysis (GSEA) (69) using the **SetRank** package in **R** (70, 71) and the Reactome pathway database (72) for the gene set definitions; and (b) over-representation analysis (ORA) using the **tmod** package (73) in R with the Reactome pathway database (72) and (c) ORA using **WebGestalt** (74). For ORA we used Fisher’s exact test to estimate the significance of enrichment for a category. We used the weighted set cover for gene set redundancy reduction, which finds a minimum subset of gene sets that include the maximum number of genes by iteratively adding sets based on a marginal benefit for adding the set. The marginal benefit is the number of genes that will be added (distinct and not yet present among all included sets) multiplied by −log_10_(p) for the set (74). **SetRank** produces a network representation of the enriched pathways that was visualized using **Cytoscape** (75).

We merged the networks from all 5 models (2.1 to 2.5) into a single network to create a master layout of all enriched pathways. The network graph of each model was then represented using the master layout to generate per-model graphs with identical positioning of pathways for ease of comparison. Analyses controlled for false discovery using either the Benjamini and Hochberg (68) or Holm (76) approach. The **SetRank** output includes two rounds of correction with the Holm procedure. Since this second correction is dependent on the collection of pathways identified as enriched after applying the first correction, it is specific to the results for a given model. We therefore present the results for the comparison of models using only the p-values adjusted with the first correction.

### Identification of Transcription Factors (TFs) and Their Binding Sites in the Promoter Regions

We downloaded 485 experimental transcription regulators (TRs) from ReMap, which includes transcription factors, transcriptional co-activator and chromatin regulators (77), generated from previous chromatin immunoprecipitation sequencing (ChIP-seq) experiments, across 346 cell types. We used **ReMap** to obtain the co-ordinates of the TF binding sites (TFBS). For each gene, we then extracted the TF binding site start (TFStart) and end (TFEnd), chromosome, TF, track, location, strand, gene transcription start site (GeneTSS), and symbol for a promoter of 4,000 bases upstream of the transcription start site of the target genes from human genome GRCh38.p13, using in-house scripts. To estimate over-representation of the TFs in PETGenes, we first extracted the TFBS and their genomic features from the list of PETGenes. Next, we calculated the counts of gene promoters containing the TFs. The counts are for gene promoters that have at least one TFBS for the TFs. Finally, we computed the TF over- or under- representation for a set of TFs comparing the frequencies in the query set (PETGenes) to that of the remaining genes in the reference (Human Genome), using Fisher’s exact test and an FDR < 0.05 (68).

### Construction of Gene Regulatory Network

We used the induced network module analysis in ConsensusPathDB (78, 79) to build a regulatory network from genes of interest. ConsensusPathDB is a database that integrates gene regulatory interaction, physical protein interactions, genetic interactions, metabolic and signaling reactions, and drug-target interactions, in human, mouse and yeast, from 30 public resources. The induced network module analysis uses regulatory information from public databases to connect genes based on their regulatory interaction. We exported the built network from ConsensusPathDB, into Cytoscape (75) for visualization. We integrated the RNA-seq expression data, to describe the functionality of the gene regulatory network at different time points. The interaction among genes in this network suggest transcriptional regulatory interaction, and their expression profiles suggest up-regulation. The feedback loops indicate gene-protein synthesis, influenced by transcription factors, i.e., a transcription factor regulates its target gene to synthesize its protein.

## Results

To identify biological processes underlying the resolution of lung inflammation, during PTB treatment, we first identified genes with significant changes in expression, over time, using Model 1. We refer to these as TimeGenes. Biological processes enriched in these genes reflect changes in transcript levels, over time, in response to PTB treatment. Next, using different forms of Model 2 (Models 2.1 to 2.5), we identified genes with expression levels significantly correlated with the changes in the PET metric MSLA, in patients who were considered cured at the end of the 6-month PTB treatment. Using GSEA, we could identify the biological processes and pathways associated with these genes that undergo changes in transcript levels in peripheral blood, and which correlated with inflammation in the lungs during PTB treatment.

### Deconvolution

**Figure 2:**
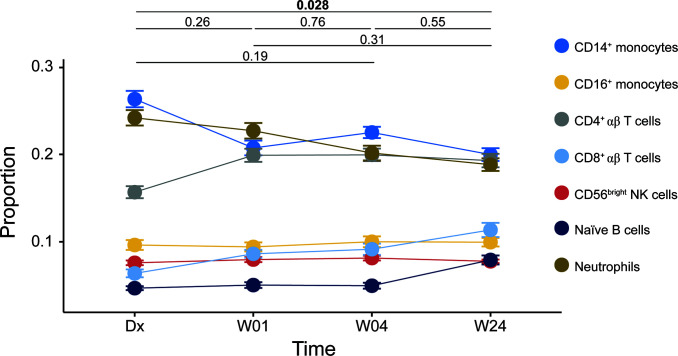
Cell proportions estimated using CIBERSORT. Line plot of repeated-measures ANOVA result. Symbols: filled circles, mean; error bars, standard error of the mean. Significance of the overall comparison between time points is indicated at the top (Tukey HSD).

We filtered cell proportions to remove cell types that did not reach a mean proportion > 0.05 at any time point. Seven cell types remained, namely CD14^+^ monocytes, CD16^+^ monocytes, CD4^+^ αβ T cells, CD8^+^ αβ T cells, CD56^bright^ NK cells, naïve B cells and neutrophils. The results from the ANOVA are shown in **Figure 2**. Four of the cell types, naïve B cells, CD8^+^ αβ T cells, CD14^+^ monocytes and neutrophils, showed changes in proportions over time in a manner that suggested that they could influence the statistical model.

### Model 1: Changes in Transcript Levels, Over Time, in Peripheral Blood During PTB Treatment (TimeGenes)

As a baseline analysis with which to compare our constrained analysis, we applied a simple model that identified transcripts that change over time. We identified 11,229 transcripts (10,295 genes, with Entrez Gene ID) with significant changes in expression levels, over time, using Model 1 (**Table S1**). We used WebGestalt and ORA to identify gene ontology biological process annotations enriched in these genes. Among the 289 significantly overrepresented gene ontology biological process annotations, we observed many related to immune response as well as annotations like “response to molecule of bacterial origin” and “cellular response to drug” (**Table S2**) emphasizing that Model 1, by not being constrained, detected all changes over time including numerous processes that do not contribute to understanding the pathophysiology of TB.

### Model 2: Changes in Transcript Levels in Peripheral Blood, Correlated With Lung [^18^F]FDG Uptake Levels (PETGenes)

We found a significant decrease in the average [^18^F]FDG uptake levels as represented by MSLA, over time, during PTB treatment (**Figure S2**). MSLA is a PET metric, previously used to calculate [^18^F]FDG lung lesion activity, and was reported to correlate with changes in lung inflammation (16, 57). To identify genes with expression levels in correlation with MSLA levels, over time, we used Model 2. We found 693 transcripts (639 with Entrez Gene ID) with significant changes in expression levels, in correlation with MSLA levels, over time (**Table S3**) using the Base model (2.1), and refer to these as PETGenes.

As expected, most of the PETGenes were among the TimeGenes, with an overlap of 591 (p=1.7e-130; odds ratio=13.8) genes between TimeGenes and PETGenes (**Table 2** and **Table S4**). Only 48 genes were new to the PETGenes and not amongst the TimeGenes, a result of intra-subject correlation between the gene expression and PET levels. The 591 overlapping genes represent changes in transcript levels in peripheral blood, associated with the resolution of inflammation in the lungs, during PTB treatment.

**Table 2:** Significance test for the overlap between TimeGenes and PETGenes.

	Not in TimeGenes	In TimeGenes
Not in PETGenes	3909	9702
In PETGenes	48	591

Genes were annotated using **biomaRt** (56). TimeGenes (n=10293); PETGenes (n=639); Intersection size=591; Overlapping p-value=2.30e-39; Odds ratio=4.96; Overlap tested using Fisher’s exact test (alternative=greater); Jaccard Index=0.06.

**Figure 3:**
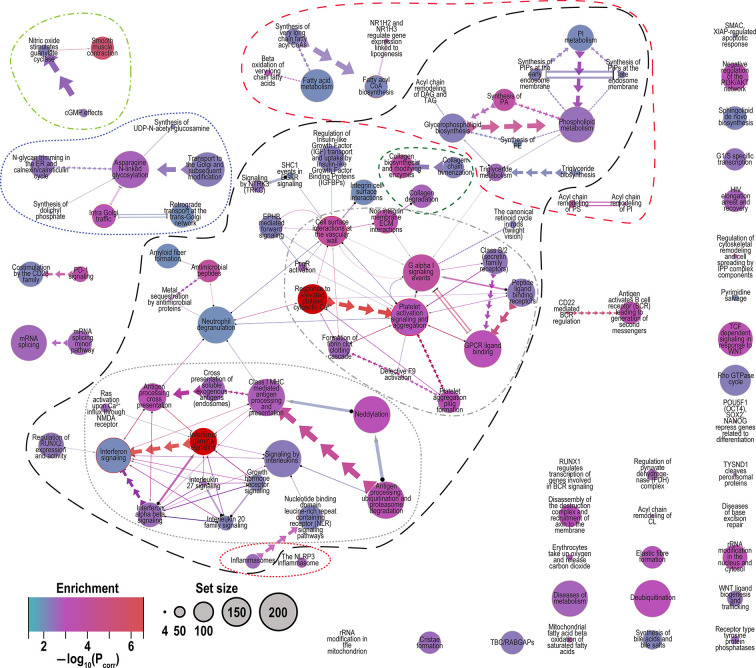
Merged network of Reactome pathways identified by all models (2.1 to 2.5). Network graphically shows the interconnectedness of many pathways through sharing of genes. Legend: Enrichment, color indicates the degree of enrichment [−log_10_(P_corr_)]; Set size, circle size represents the number of genes in the pathway conditioned on the observed 14,841 genes. Edge (connector) symbols: straight line, limited gene sharing; arrows, subset; and double lines, substantial overlap; line thickness indicates degree of gene sharing [−log_10_(P_intersect_)]. Related pathways are indicated by the outlines. Long black dashes, the largest interconnected set of pathways comprising “interferon and interleukin signaling”, “platet activation and degranulation” and “phospholipid metabolism”; dotted red, inflammasomes; dotted grey, interferon and interleuking signaling; dash-dot grey, platelet activation and degranulation; short-dash green, collagen metabolism; medium dash red, lipid and phospholipid metabolism; dot-dash light green, smooth muscle contraction; and dotted dark blue, Golgi trafficking and N-linked glycosylation. Note: in the merged network the pathway colors are represented as the minimum corrected P value [or maximum −log_10_(P_corr_)] among the five models.

We performed GSEA using the model fit results per gene and **SetRank** with the Reactome pathways (72). We identified 47 pathways that were enriched for the Base model (2.1) (**Table 3** and **Figure S3**). Models 2.2 to 2.5 identified 48, 35, 35 and 33 pathways, respectively, as enriched (**Table 4**). Overall, we identified 103 Reactome pathways (72) as enriched in one or more of the models (**Figures 3**, **S3** **–** **S7** and **Table 4**). Many of the well-known pathways in TB biology were represented among the enriched pathways (80), but the results also included some novel observations. Nine pathways were identified in all models: “Interferon gamma signaling”, “Response to elevated platelet cytosolic Ca^2+^”, “Smooth muscle contraction”, “G alpha I signaling events”, “Platelet activation signaling and aggregation”, “Cell surface interactions at the vascular wall”, “rRNA modification in the nucleus and cytosol”, “PD-1 signaling” and “Interferon signaling” (**Table 4**).

**Table 3:** Reactome pathways enriched by GSEA using SetRank for the base model (not corrected for cell proportion).

Pathway	Size	Set Rank	Corrected P-Value*	Adjusted P-Value^†^
Platelet activation signaling and aggregation	229	0.0753	1.12E-04	5.93E-10
Response to elevated platelet cytosolic Ca2+	114	0.0493	2.28E-11	5.93E-10
Interferon gamma signaling	82	0.0480	3.95E-11	5.93E-10
Interferon alpha beta signaling	56	0.0259	1.44E-07	5.93E-10
Antimicrobial peptides	32	0.0192	1.38E-04	5.93E-10
Cell surface interactions at the vascular wall	117	0.0372	2.16E-04	5.93E-10
Non integrin membrane ECM interactions	47	0.0223	4.02E-04	5.93E-10
Cross presentation of soluble exogenous antigens (endosomes)	48	0.0121	5.54E-04	5.93E-10
Antigen processing cross presentation	95	0.0141	6.88E-04	5.93E-10
G alpha I signaling events	248	0.0301	8.14E-04	5.93E-10
Interferon signaling	175	0.0259	9.42E-04	5.93E-10
GPCR ligand binding	243	0.0206	9.54E-04	5.93E-10
Defective F9 activation	4	0.0121	9.71E-04	5.93E-10
Platelet aggregation plug formation	31	0.0121	1.01E-03	5.93E-10
Collagen degradation	43	0.0121	1.95E-03	5.93E-10
Interleukin 27 signaling	10	0.0141	2.01E-03	5.93E-10
Metal sequestration by antimicrobial proteins	4	0.0121	2.37E-03	5.93E-10
Class B/2 (secretin family receptors)	62	0.0121	3.85E-03	5.93E-10
Peptide ligand binding receptors	97	0.0121	4.20E-03	5.93E-10
The canonical retinoid cycle in rods (twilight vision)	11	0.0121	4.41E-03	5.93E-10
Regulation of insulin like growth factor (IGF) transport and uptake by insulin like growth factor binding proteins (IGFBPs)	79	0.0121	5.20E-03	5.93E-10
Growth hormone receptor signaling	21	0.0171	5.88E-03	5.93E-10
Interleukin 20 family signaling	19	0.0121	6.10E-03	5.93E-10
Neutrophil degranulation	457	0.0121	9.92E-03	5.93E-10
Amyloid fiber formation	58	0.0121	9.93E-03	5.93E-10
Smooth muscle contraction	33	0.0223	1.28E-07	3.19E-06
cGMP effects	12	0.0121	5.11E-03	3.19E-06
Nitric oxide stimulates guanylate cyclase	16	0.0121	6.97E-03	3.19E-06
CD22 mediated BCR regulation	5	0.0121	9.21E-06	2.21E-04
Antigen activates B cell receptor (BCR) leading to generation of second messengers	29	0.0121	2.82E-04	2.21E-04
Erythrocytes take up oxygen and release carbon dioxide	9	0.0121	2.05E-04	4.71E-03
PD-1 signaling	20	0.0121	2.32E-04	5.11E-03
Costimulation by the CD28 family	65	0.0121	6.84E-03	5.11E-03
Regulation of cytoskeletal remodeling and cell spreading by IPP complex components	8	0.0121	4.64E-04	9.75E-03
Acyl chain remodeling of PS	11	0.0223	5.24E-04	1.05E-02
Acyl chain remodeling of PI	7	0.0121	5.24E-04	1.05E-02
rRNA modification in the nucleus and cytosol	59	0.0121	5.84E-04	1.11E-02
Diseases of base excision repair	5	0.0121	9.89E-04	1.78E-02
Elastic fibre formation	34	0.0121	1.18E-03	2.00E-02
Synthesis of very long chain fatty acyl CoAs	21	0.0121	1.84E-03	2.94E-02
RUNX1 regulates transcription of genes involved in BCR signaling	6	0.0121	2.02E-03	3.03E-02
Inflammasomes	19	0.0121	2.49E-03	3.49E-02
Receptor type tyrosine protein phosphatases	12	0.0121	2.55E-03	3.49E-02
Cristae formation	31	0.0121	2.65E-03	3.49E-02
Asparagine N-linked glycosylation	281	0.0121	2.87E-03	3.49E-02
rRNA modification in the mitochondrion	5	0.0121	3.08E-03	3.49E-02
SMAC, XIAP-regulated apoptotic response	8	0.0121	4.76E-03	4.28E-02

*Holm correction for multiple testing; ^†^Holm correction 2^nd^ round, correcting for dependence among pathways.

Size indicates the number of genes in the pathway conditioned on the genes detected as transcribed.

**Table 4:** Reactome pathways enriched by GSEA using SetRank for all models.

Pathway Name	Correcting for 3 cell types				
	Base*	B Cell*	CD8 T Cell*	Monocyte*	Neutrophil*
Interferon gamma signaling	**3.95E-11**	8.64E-10	9.52E-09	4.94E-09	2.03E-09
Response to elevated platelet cytosolic Ca^2+^	**2.28E-11**	2.33E-09	7.72E-06	6.90E-07	1.43E-06
Smooth muscle contraction	**1.28E-07**	1.40E-07	2.40E-05	9.34E-06	3.28E-06
G alpha I signaling events	**8.14E-04**	2.81E-04	2.20E-04	1.32E-04	1.61E-04
Platelet activation signaling and aggregation	**1.12E-04**	1.19E-03	2.56E-03	2.64E-04	3.80E-03
Cell surface interactions at the vascular wall	**2.16E-04**	2.96E-04	3.45E-03	8.58E-04	2.12E-03
rRNA modification in the nucleus and cytosol	**5.84E-04**	3.49E-04	1.34E-03	5.84E-04	1.10E-03
PD-1 signaling	**2.32E-04**	3.93E-04	5.51E-04	3.57E-04	3.57E-04
Interferon signaling	**9.42E-04**	8.17E-04	4.50E-03	3.19E-03	8.51E-03
Antigen processing: ubiquitination and proteasome degradation		1.99E-04	**1.04E-04**	1.99E-03	6.35E-04
Cross presentation of soluble exogenous antigens (endosomes)	**5.54E-04**		1.53E-04	1.53E-04	2.27E-03
Collagen biosynthesis and modifying enzymes		2.36E-04	**2.19E-04**	9.13E-04	3.10E-04
Class I MHC mediated antigen processing and presentation		3.23E-04	**2.31E-04**	2.31E-04	1.56E-03
Defective F9 activation	**9.71E-04**	3.74E-04		8.17E-03	8.37E-03
Asparagine N-linked glycosylation	**2.87E-03**		9.78E-04	9.61E-03	1.85E-03
POU5F1 (OCT4), SOX2, NANOG repress genes related to differentiation		4.36E-03	1.42E-03	**1.04E-03**	1.24E-03
Interferon alpha beta signaling	**1.44E-07**	5.23E-07			2.70E-03
EPHB mediated forward signaling			**1.60E-05**	6.04E-03	1.70E-03
Glycerophospholipid biosynthesis		**2.39E-05**	2.02E-03	5.27E-03	
Synthesis of PA			7.21E-04	1.98E-03	**2.13E-04**
Neddylation			**8.83E-04**	4.27E-04	6.54E-04
Diseases of base excision repair	**9.89E-04**	2.15E-03		1.63E-03	
N-glycan trimming in the ER and calnexin/calreticulin cycle			**3.78E-03**	5.78E-03	8.19E-03
Synthesis of dolichyl phosphate			9.06E-03	**5.00E-03**	7.09E-03
Synthesis of very long chain fatty acyl CoAs	**1.84E-03**	5.84E-03			5.38E-03
Nitric oxide stimulates guanylate cyclase	**6.97E-03**	5.82E-03			9.55E-03
CD22 mediated BCR regulation	**9.21E-06**	3.29E-04			
Antigen activates B cell receptor (BCR) leading to generation of second messengers	**2.82E-04**	4.85E-04			
TCF dependent signaling in response to WNT			**6.01E-04**		1.65E-03
Inflammasomes	**2.49E-03**	6.57E-04			
TBC/RABGAPs		4.51E-03		**9.03E-04**	
Regulation of cytoskeletal remodeling and cell spreading by IPP complex components	**4.64E-04**	1.00E-03			
Intra Golgi traffic		9.76E-03		**1.13E-03**	
Erythrocytes take up oxygen and release carbon dioxide	**2.05E-04**	1.23E-03			
Fatty acid metabolism		**1.30E-03**	8.86E-03		
FcgR activation		4.85E-03		**1.62E-03**	
Regulation of pyruvate dehydrogenase (PDH) complex		**7.81E-03**			2.05E-03
cGMP effects	**5.11E-03**	4.26E-03			
Synthesis of UDP-N-acetyl-glucosamine			**4.82E-03**		6.58E-03
Synthesis of PE			**5.33E-03**	1.00E-02	
RUNX1 regulates transcription of genes involved in BCR signaling	**2.02E-03**	7.66E-03			
Neutrophil degranulation	**9.92E-03**	7.71E-03			
SMAC, XIAP-regulated apoptotic response	**4.76E-03**	8.48E-03			
The canonical retinoid cycle in rods (twilight vision)	**4.41E-03**	9.36E-03			
Negative regulation of the PI3K/AKT network		6.85E-04			
Mitochondrial fatty acid beta oxidation of saturated fatty acids				8.49E-04	
Disassembly of the destruction complex and recruitment of axin to the membrane				9.02E-04	
Deubiquitination				9.25E-04	
Acyl chain remodeling of CL					9.83E-04
Beta oxidation of very long chain fatty acids			1.21E-03		
NR1H2 and NR1H3 regulate gene expression linked to lipogenesis					1.35E-03
HIV elongation arrest and recovery			1.79E-03		
The NLRP3 inflammasome				1.93E-03	
Diseases of metabolism		2.04E-03			
mRNA splicing minor pathway				2.16E-03	
mRNA splicing				2.29E-03	
Formation of fibrin clot clotting cascade		2.30E-03			
Ras activation upon Ca^2+^ influx through NMDA receptor			2.37E-03		
Triglyceride metabolism			2.42E-03		
TYSND1 cleaves peroxisomal proteins		2.59E-03			
Phospholipid metabolism			3.14E-03		
WNT ligand biogenesis and trafficking		3.58E-03			
G1/S specific transcription		3.61E-03			
Regulation of RUNX2 expression and activity					3.83E-03
Signaling by NTRK3 (TRKC)					4.09E-03
Signaling by interleukins		4.78E-03			
Transport to the Golgi and subsequent modification			5.20E-03		
Rho GTPase cycle		5.41E-03			
Acyl chain remodeling of DAG and TAG				5.50E-03	
Collagen chain trimerization		5.96E-03			
Synthesis of bile acids and bile salts		6.35E-03			
Nucleotide binding domain leucine-rich repeat containing receptor (NLR) signaling pathways		6.42E-03			
Synthesis of PIPs at the early endosome membrane			6.57E-03		
Synthesis of PIPs at the late endosome membrane			6.57E-03		
Sphingolipid *de novo* biosynthesis		7.19E-03			
Fatty acyl CoA biosynthesis					7.39E-03
SHC1 events in EGFR signaling					8.26E-03
PI metabolism			8.29E-03		
Pyrimidine salvage		8.89E-03			
Triglyceride biosynthesis			9.35E-03		
Integrin cell surface interactions				9.47E-03	
Retrograde transport at the trans-Golgi network		9.76E-03			
GPCR ligand binding	9.54E-04				
Antimicrobial peptides	1.38E-04				
Interleukin 27 signaling	2.01E-03				
Acyl chain remodeling of PS	5.24E-04				
Non integrin membrane ECM interactions	4.02E-04				
Regulation of Insulin-like Growth Factor (IGF) transport and uptake by Insulin-like Growth Factor Binding Proteins (IGFBPs)	5.20E-03				
Peptide ligand binding receptors	4.20E-03				
Antigen processing cross presentation	6.88E-04				
Acyl chain remodeling of PI	5.24E-04				
Amyloid fiber formation	9.93E-03				
Elastic fibre formation	1.18E-03				
Platelet aggregation plug formation	1.01E-03				
Cristae formation	2.65E-03				
Growth hormone receptor signaling	5.88E-03				
rRNA modification in the mitochondrion	3.08E-03				
Receptor type tyrosine protein phosphatases	2.55E-03				
Class B/2 (secretin family receptors)	3.85E-03				
Collagen degradation	1.95E-03				
Metal sequestration by antimicrobial proteins	2.37E-03				
Interleukin 20 family signaling	6.10E-03				
Costimulation by the CD28 family	6.84E-03				

*Corrected p-value (P_corr_) value using Holm correction for multiple testing [since the **SetRank** second round of Holm correction (P_adj_) is dependent on the specific collection of pathways, results are presented using P_corr_); Bold p-values, minimum p-value for rows with more than one p-value. Models: Base, model 2.1; B Cell, model 2.2; CD8 T Cell, model 2.3; Monocyte, model 2.4; and Neutrophil, model 2.5 (for details see **Methods**).

Many pathways are related by sharing of genes and therefore function, but some of the other pathways can be grouped together by related function as shown in **Figure 3**. Approximately half of the pathways represented are connected (outlined in long black dashed line, **Figure 3**). The inter-related pathways include “platelet activation, signaling and aggregation”, “extracellular matrix reorganization”, “cell surface interactions at the vascular wall” and “response to elevated cytosolic Ca^2+^” (grey dot-dash **Figure 3**) (80, 81), the interferon and interleukin signaling cluster (grey dotted **Figure 3**) (80, 81), and a cluster of phospholipid acyl chain metabolism that includes unconnected small clusters of lipid metabolism pathways (red medium dash **Figure 3**) (82). Smaller clusters of pathways include Golgi trafficking (83) and N-glycosylation (dotted dark blue **Figure 3**) (84), and “smooth muscle contraction” (light green dot-dash, **Figure 3**).

**Figure 4:**
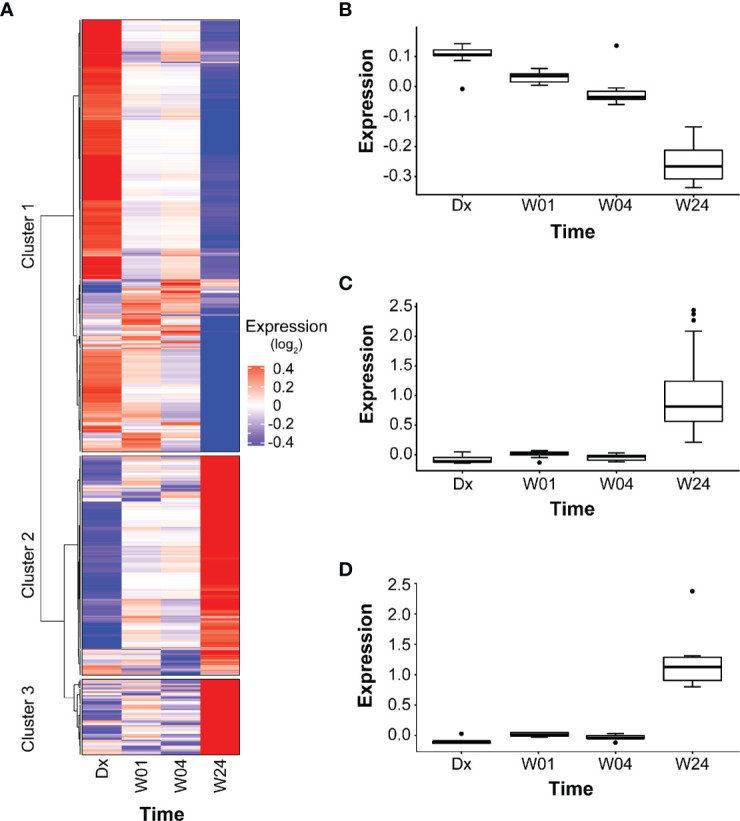
Clusters in PETGenes. **(A)** Co-expression of PETGenes grouped into clusters. Each cluster contains genes with similar expression pattern, over time. Dark blue, very low expression level; grey, moderate expression level; and dark red, very high expression level. Expression (log_2_), average expression of 75 subjects at each time point. **(B)** Changes in the expression of Cluster 1 genes. **(C)** Changes in the expression of Cluster 2 genes, and **(D)** Changes in the expression of Cluster 3 genes.

Among the 639 PETGenes identified in Model 2.1, we found 3 distinct clusters of genes, with similar expression patterns over time (co-expression) (**Figure 4A**, **Table S5**). The expression levels of PETGenes in Cluster 1 started high at Dx, and decreased over time (378 genes; **Figure 4B**) whereas genes in Cluster 2 (191 genes; **Figure 4C**) and Cluster 3 (66 genes; **Figure 4D**), started low at Dx and increased over time (**Figure 4D**), thus suggesting up-regulation in Clusters 2 and 3 (**Figures 4C, D**), and down-regulation in Cluster 1 (**Figure 4B**).

Pathways related to platelets in Cluster 1 and B cells in Cluster 3 were significantly overrepresented (**Table 5**, **Table S5**). Some new pathways in Cluster 1 included “hemostasis”, “adaptive immune system”, “innate immune system”, “p130Cas linkage to MAPK signaling for integrins”, and “GRB2 SOS provides linkage to MAPK signaling for integrins”. The modules enriched in B cells included the following PETGenes in Cluster 3: *CD19, VPREB3, CD72, CD22, FCRLA, CD79B, CD79A, HLA-DOB, P2RX5, FAM129C, FCRL5, PCDH9, BCL11A, PNOC*, and *BLNK.* The expression profiles of PETGenes in Cluster 3, suggest up-regulation in B cells during treatment response, whereas Cluster 1, suggests down-regulation in platelet activation during treatment response. Curiously, there were no pathways overrepresented in Cluster 2 (**Figure 4C**).

**Table 5:** Overrepresented Reactome pathways in clusters of PETGenes.

Pathway	b		B	E	FDR
**Cluster 1**					
Hemostasis	53	512		3.98	7.12E-15
Platelet activation signaling and aggregation	34	228		5.73	9.46E-14
Response to elevated platelet cytosolic Ca^2+^	21	113		7.14	6.26E-10
Interferon signaling	23	175		5.05	6.45E-08
Interferon gamma signaling	16	82		7.50	8.80E-08
Cytokine signaling in immune system	46	728		2.43	5.74E-06
Formation of fibrin clot clotting cascade	8	28		10.98	8.56E-05
Interferon alpha beta signaling	10	56		6.86	3.02E-04
Cell surface interactions at the vascular wall	14	117		4.60	3.60E-04
Innate immune system	47	893		2.02	4.86E-04
Platelet aggregation plug formation	7	31		8.68	0.0017
Smooth muscle contraction	7	33		8.15	0.0024
G alpha I signaling events	19	248		2.94	0.0033
Antigen processing cross presentation	11	95		4.45	0.0039
Adaptive immune system	37	705		2.02	0.0041
Signaling by GPCR	29	524		2.13	0.0110
Class A/1 rhodopsin like receptors	14	171		3.15	0.0142
GRB2 SOS provides linkage to MAPK signaling for integrins	4	12		12.81	0.0155
p130Cas linkage to MAPK signaling for integrins	4	12		12.81	0.0155
Regulation of insulin like growth factor (IGF) transport and uptake by insulin like growth factor binding proteins (IGFBPs)	9	79		4.38	0.0160
GPCR ligand binding	17	243		2.69	0.0161
Common pathway of fibrin clot formation	4	14		10.98	0.026
Signaling by interleukins	22	380		2.22	0.028
Nitric oxide stimulates guanylate cyclase	4	15		10.25	0.032
Intrinsic pathway of fibrin clot formation	4	16		9.61	0.037
Cell extracellular matrix interactions	4	16		9.61	0.037
Interleukin 4 and interleukin 13 signaling	9	92		3.76	0.037
Extracellular matrix organization	15	220		2.62	0.037
Cell-cell communication	9	96		3.60	0.045
Cross presentation of particulate exogenous antigens (phagosomes)	3	8		14.41	0.045
Regulation of cytoskeletal remodeling and cell spreading by IPP complex components	3	8		14.41	0.045
Peptide ligand binding receptors	9	97		3.57	0.046
**Cluster 3**					
CD22-mediated B cell receptor (BCR) regulation	3	5		278.10	1.41E-04
Antigen activates B cell receptor (BCR) leading to generation of second messengers	4	29		63.93	3.17E-04
Signaling by the B cell receptor (BCR)	4	107		17.32	0.041

N = 14832; Cluster 1 n = 378; Cluster 3 n = 66.

Columns: b, number of genes in the gene set/pathway and in the cluster (n); B, number of genes in the gene set and in the observed genes (N); E, enrichment score; FDR, false discovery rate.

To validate the PETGenes, we compared our results against published gene signatures of TB treatment response and transcriptional profiling of lung biopsies from PTB subjects, using Fisher’s exact test. We found 63 PETGenes overlapped (p=7.3e-34; odds ratio=8.34) with the treatment-specific signature from Bloom et al. (24) (320 microarray probes mapping to 267 distinct protein-coding genes; **Table S6**), and 99 PETGenes overlapped (p=3.8e-136; odds ratio=576.1) with the 393-probe, 307-gene, signature from Berry et al. (22) (**Table S7**). Three PETGenes, *ITGB3, MMP1*, and *STAT1* were found to overlap with “top 15 most highly differentially expressed regulator genes in lung TB granulomas” from Subbian et al. (85), 29 genes overlapped (p=7.1e-21; odds ratio=13.7) with “TB blood biomarkers in the lung” from Subbian et al. (85) (**Table S8**), and although 164 overlapped with the “list of significantly differentially expressed genes common to fibrotic nodules and cavitary lesions” from Subbian et al. (85) (**Table S9**), this was not significant due to the size of the set (4,417 genes) (p=0.99; odds ratio=0.79). Additionally, we investigated if there was an overlap between the 639 genes identified here and prior small signatures (27). Due to the size of the signatures, we did not perform tests for significance of overlap. We identified 17 prior signatures (**Table S10**). Of these 3 were for conditions that are not relevant to the current study, i.e., prediction of failure, response to treatment, and diagnosis of active TB from latent TB. The subjects in this study were not failures, were all cured at end of treatment, and there were no latent TB cases. After removing these three signatures, the mean proportion of genes shared was 0.60 (**Table S10**).

### Identification of Transcriptional Regulatory Factors in PETGenes

We found 254 TFs to be significantly over-represented in the list of PETGenes, with FDR < 0.05 (**Table S11**). Among the over-represented TFs in PETGenes (**Table S11**), *NF-KB1, CREB, STAT, FOS*, and *JUN*, have been reported to play a critical role in regulating inflammation in lung diseases (86), and we extracted their TFBS in the promoter region of their target genes (**Table S12**).

Further, we identified an induced regulatory network among Cluster 3 genes enriched for B cell genes and pathways. The expression level of genes in this network was low at Dx (**Figure S8**), and high at W24 (**Figure S9**). The induced gene network suggests transcriptional regulation of B cell genes to synthesize proteins (**Figures S8**, **S9**). The genes in this network were enriched in “B cell activation” and “B cell receptor signaling pathway”, thus suggesting up-regulation in B cell activation. Among Cluster 3 genes, we identified genes coding for TFs *UBTF, MITF, ATF3, GRHL1, SPIB, STAT1*, and *STAT2*. We extracted their TFBS (see ***Methods***), as well as their target genes (**Table S12**). The TFs *PAX5, EBF1*, and *SPIB* are included in Cluster 3, and *CD19, CD79A*, and *BLK* are direct regulatory targets of *PAX5* (**Figure S8**, **Table S12**)*. PAX5* and *EBF1* have been reported to regulate the expression of B cell genes and play an important role in B cell development (87, 88). Gene set enrichment revealed that B cell receptor signaling pathway is significantly enriched in Cluster 3 genes: *PAX5, SPIB, EBF1, CD19, CD79A, BLK, BLNK, CD79B, CD22*, and *CD72* (31).

We identified a regulatory network among Cluster 1 genes enriched in genes related to platelet activation and signaling. The expression profiles of the genes included in this network were high at Dx and low at W24 (**Figure S10**, and **Figure S11**, respectively). The following genes were significantly enriched in platelet degranulation: *F13A1, ITGA2B, ITGB3, LY6G6F, PF4, PPBP, RAB27B, SERPINE1*, and *STTL4.* The KEGG pathway “platelet activation” was significantly enriched in Cluster 1 genes: *F2RL3, GP1BA, GP6, GP9, ITGA2B, ITGB3, PRKG1*, and *PTGS1.* Also, we observed high expression level in platelet surface markers: *GP9, GP1BA, GP6, PF4, GP1BB, ITGB3, ITGA2B, F2RL3*, and *ITGB5* at Dx (**Figure S10**), and low expression at W24 (**Figure S11**), thus suggesting down-regulation of platelet genes. We identified a complex of interacting platelet surface receptors, which exhibited high expression at Dx (**Figure S10**), suggesting functional activation of platelets at Dx, and low expression at W24 (**Figure S11**), suggesting down-regulation of platelet activation during treatment.

In addition, we observed interaction between tissue factor pathway inhibitor (TFPI) and *MMP1, MMP8*, and *MMP12*, which were enriched with the biological processes: “coagulation” and “regulation in response to wounding”, respectively. These genes exhibited high expression at Dx (**Figure S10**), and low expression at W24 (**Figure S11**), suggesting down-regulation of *MMP-*driven coagulation.

**Figure 5:**
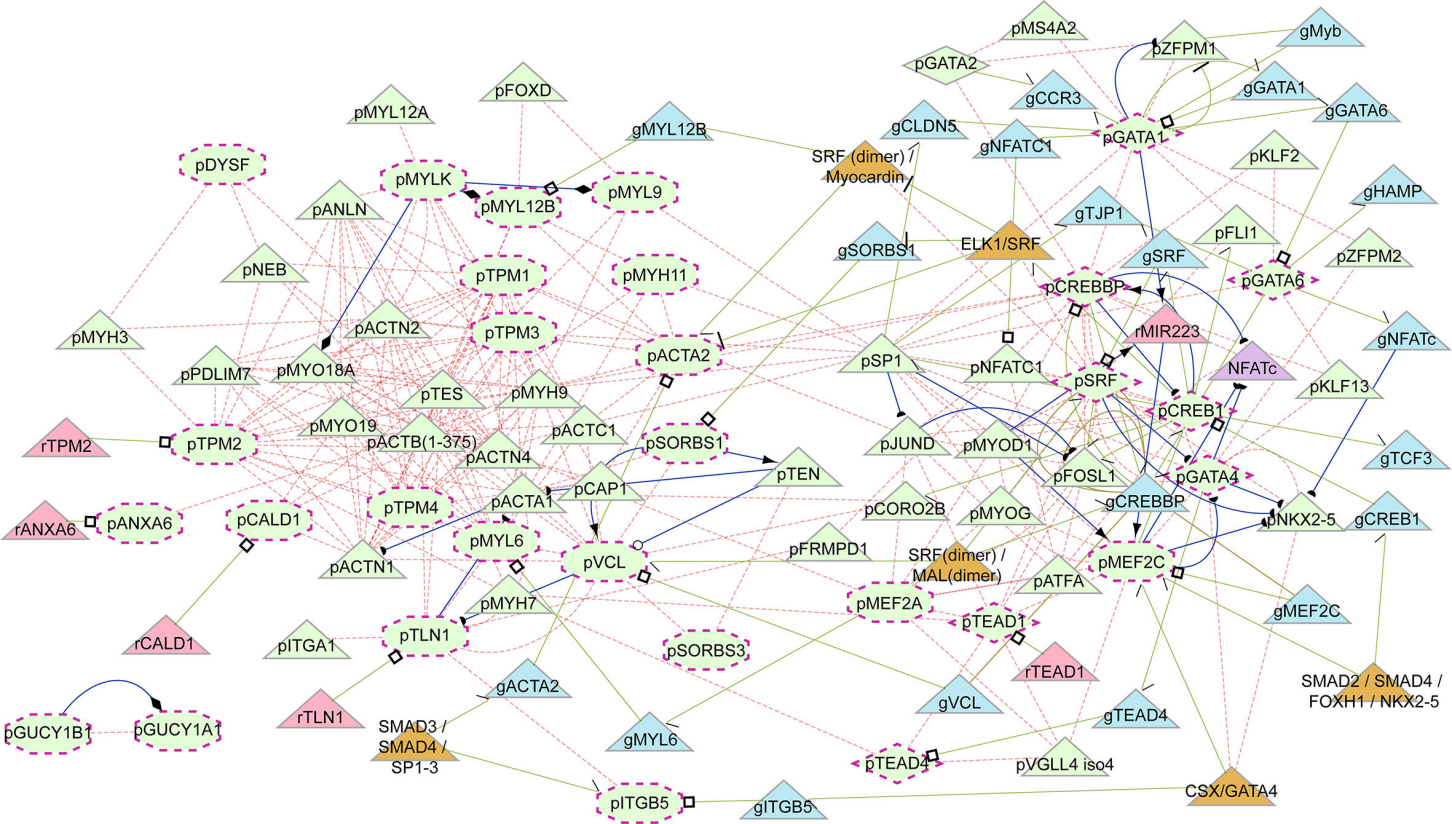
Induced regulatory network of smooth muscle cell contraction. Network generated from genes enriched in the smooth muscle contraction pathway together with enriched transcription factors present in their promoters. Shapes: maroon dashed-line border, input elements; octagons, input smooth muscle genes (as proteins); diamonds, input transcription factors (as proteins); triangles, induced elements; light green elements, proteins; light blue, genes; light red, RNA; orange, complexes. Text prefixes: g, gene; p, protein; r, RNA. Lines: light red dashed, protein interaction; light green, gene regulatory interaction; blue, biochemical interaction. Target arrow shapes: arrowhead, activator; open circle, product, half circle, substrate; diamond, enzyme activity; T, suppressor; open square, transcribed product; half arrow/half top, transcription factor binding.

Smooth muscle contraction was identified by GSEA as well as by ORA among Cluster 1 genes. Cluster 1 genes decrease in expression from Dx to W24 (**Figure 4B**), suggesting that the pathway is downregulated during resolution of inflammation. We verified that 15 of the 33 genes in the pathway have mean transcript levels in excess of 100 transcripts per million, and most of the genes are transcribed above the nominal 10 transcripts per million (after adjusting for library size). Additionally, it should be noted that the transcription level is expected to be much higher in the sub-population of cells in peripheral blood that express the smooth muscle contraction phenotype. We constructed a gene regulatory network using the genes in the smooth muscle contraction pathway as well as the subset of transcription factors identified as enriched that also occurred in the smooth muscle contraction promoters (**Figure 5**). The genes enriched in Cluster 1 were: *CALD1*, *GUCY1A1*, *GUCY1B1*, *ITGB5*, *MYL9*, *TPM1*, *TPM4*, *VCL*. To these we added the genes whose nominal p-value contributed to the GSEA enrichment score: *ACTA2*, *ANXA6*, *DYSF*, *MEF2A*, *MEF2C*, *MYH11*, *MYL12B*, *MYL6*, *MYLK*, *SORBS1*, *SORBS3*, *TLN1*, *TPM2*, *TPM3*, as well as the transcription factors: *CREB1*, *CREBBP*, *GATA1*, *GATA2*, *GATA4*, *GATA6*, *SRF*, *TEAD1*, *TEAD4*. All but *SORBS1*, *SORBS3*, *TPM2*, and *VCL* decreased expression from Dx to W24. The network demonstrates that a highly interconnected cluster of contraction proteins connected to another interconnected cluster of transcription factors (**Figure 5**).

## Discussion

In this study using linear mixed-effects models accommodating intra-subject correlation between outcome and explanatory variables, we have identified 639 genes which exhibit changes in blood transcript levels that correlate with [^18^F]FDG uptake changes in lung lesions during successful PTB treatment. These 639 genes are almost entirely a subset of the 10,295 genes that change over time during treatment. Since all modelled subjects were clinically cured at the end of treatment, the genes identified by Model 2 are those correlated with resolution of lung inflammation during PTB treatment. The changes are, however, those in the peripheral blood that reflect the resolution of lung inflammation, and not the changes *in situ*. These PETGenes also allowed us to identify a number of biological processes associated with inflammation.

We identified 47 enriched pathways (**Table 3**) using a model without correction for cell proportion (Base, model 2.1), and a total of 103 enriched pathways when accounting for the influence of cell proportions, of which the 47 are a subset, in one or more of the 5 models (models 2.1 to 2.5). The correlation of expression of genes identified by models correcting for cell proportions could then be due to: a) the change in proportion of the remaining cell type; b) change in regulation of gene expression in any of the cell types; c) a change in proportion of another cell type not estimated by deconvolution; or d) a combination of the above. Since the proportions of cell types were estimated by deconvolution, the adjustment might be neither efficient, in statistical terms, nor as accurate as differential counts. Therefore, caution needs to be used in the interpretation of the results obtained here.

We identified several pathways, “CD22 mediated BCR regulation”, “antigen activates B cell receptor (BCR) leading to generation of second messengers”, and “RUNX1 regulates transcription of genes involved in BCR signaling,” whose function is restricted to B cells (**Table 4**) in model 2.2 “B Cell”. This supports the premise of the approach that adjusting for the proportions of several other cell types permits cautious attribution of at least some of the gene expression changes to corresponding changes in the proportions of the remaining cell type (B cells).

Eleven pathways, all consistent with different aspects of lipid and phospholipid metabolism are enriched in the model 2.3 “CD8 T cell.” The pathways are: synthesis of phosphatidic acid (PA); synthesis of dolichyl phosphate; fatty acid metabolism; synthesis of phosphatidylethanolamine (PE); beta oxidation of very long chain fatty acids; triglyceride metabolism; phospholipid metabolism; synthesis of phosphatidylinositol phosphates (PIPs) at the early endosome membrane; synthesis of PIPs at the late endosome membrane; PI metabolism; and triglyceride biosynthesis. Lipid metabolism has been established as important to the function of T cells (89), again supporting attribution of some of the effect to changes in proportions of the remaining cell type (CD8^+^ αβ cells). There are also several pathways identified here as potentially relevant to T cells that have not previously been identified. These include some of the phospholipid metabolism pathways and the PIP pathways.

For CD14^+^ monocytes and neutrophils no pathway or collection of pathways provided support in the same way as for naïve B cells and CD8^+^ αβ cells. The pathways identified are generally consistent with inflammatory processes, but not characteristic of the cell types.

Nine pathways were identified in all models: Interferon gamma signaling; Response to elevated platelet cytosolic Ca^2+^; Smooth muscle contraction; G alpha I signaling events; Platelet activation signaling and aggregation; Cell surface interactions at the vascular wall; rRNA modification in the nucleus and cytosol; PD-1 signaling; and Interferon signaling (**Table 4**). It seems therefore that the proportions of the 4 cell types are not relevant to their detection. These pathways could be driven by regulation in all 4 cell types, or by cells of another type, or both. Seven of the nine pathways, “Interferon gamma signaling”, “Response to elevated platelet cytosolic Ca^2+^”, “G alpha I signaling events”, “Cell surface interactions at the vascular wall”, “Platelet activation signaling and aggregation”, “PD-1 signaling”, and “Interferon signaling”, are well-established inflammatory response pathways and it is reasonable that regulation in all 4 cell types occurs during response to treatment in TB.

The remaining two pathways are: “Smooth muscle contraction,” and “rRNA modification in the nucleus and cytosol.” Although smooth muscle contraction has been identified as a pathway enriched in TB transcriptomics, it was in the context of lymph node *M. tuberculosis* infection (90). rRNA modification in the nucleus and cytosol has not been identified previously. Here we see both pathways in all models, suggesting that these are important in the biological changes that occur during response to treatment.

“Smooth muscle contraction” is an interesting pathway to detect in peripheral blood. Most of the genes characteristic of this pathway are not expressed in leukocytes. Nevertheless, it is one of the most enriched pathways in our analysis (P_corr_ < 1.3 x 10^−7^; **Table 4**). Although it has been observed before in the context of TB (90, 91), it was not interpreted or discussed. In the mouse study (91) it was observed in peripheral blood, but in the human study (90) it was in a TB-infected lymph node. Prolonged and coordinated expression of smooth muscle contraction genes in peripheral blood is unusual. This raises the question about the origin of the cells that give rise to this expression. One possibility is proliferation of smooth muscle progenitor cells (SPCs), which have been observed in peripheral blood, in response to vascular or tissue injury, for tissue repair and remodeling. SPCs have previously been identified in human and animal peripheral blood (92–94). Among the responses to injury and inflammation are signals that stimulate SPC division and migration from tissue reservoirs or the bone marrow to the site of injury (95). Platelets have been shown to interact with SPCs in peripheral blood, where platelets express adhesion receptors that enable them to interact with endothelial cells, leukocytes and SPCs (92). This interaction triggers the following in SPCs: mobilization of progenitor cells from either the bone marrow to peripheral circulation or from local tissue reservoirs, e.g., pericytes, chemotaxis to target tissue, adhesion on vascular wall, survival and engraftment into local tissue, differentiation into mature functional cell types, such as endothelial cells, and proliferation, which are essential steps of progenitor cell-mediated tissue repair (96). The extensive activation of platelets and platelet related pathways observed in TB (**Figure 3** and **Figures S3** **–** **S7**) (80, 81) suggest that mobilization of SPCs is likely. Almost all the genes in the “smooth muscle contraction” pathway are down-regulated during response to treatment consistent with overall wound-healing and a decreased requirement for angiogenesis, neo- or revascularization. A critical question for TB is whether the angiogenesis around granulomas is as dysregulated as around cancer tumors (97). The dysregulated angiogenesis around tumors prevents immune cells from crossing into the tumor and contributes to the inability of the immune system to attack the tumor (97). This could have implications for TB treatment.

Another enriched pathway “rRNA modification in the nucleus and cytosol” has previously been reported in peripheral blood of Friedrich’s Ataxia patients (98), but not in TB. It can be involved in at least two aspects. First, modification of rRNA plays a role in stability of ribosomes (99) and their efficiency in translation (99, 100). In part it is likely a response to the increased biosynthesis necessary for the immune cells in lung inflammation and wound healing. Second, modification of rRNA is also involved in progenitor cell differentiation (101). The “rRNA modification in the nucleus and cytosol” pathway could therefore tie back in to the SPC differentiation. The origins of the signal for rRNA modification can also arise in other cell types and the alterations are important for ribosome stability and efficiency (100). It is conceivable that rRNA modifications are necessary during the chronic lung inflammation of PTB and that the modifications return to homeostatic conditions when the inflammation subsides.

The cluster of mitochondrion-related pathways is also interesting. The genes are mostly downregulated during response to treatment. The pathway of “cristae” formation includes genes for proteins that induce the folding of the inner mitochondrial membrane (*MICOS10*, *MICOS13*, *DNAJC11*) and mitochondrial morphogenesis (*TMEM11*), but also includes many genes for mitochondrial complex V, the ATP synthase complex (*ATP5F1A*, *ATP5F1B*, *ATP5F1C*, *ATP5F1D*, *ATP5F1E*, *ATP5MC1*, *ATP5MC2*, *ATP5MC3*, *ATP5ME*, *ATP5MF*, *ATP5MG*, *ATP5PB*, *ATP5PD*, *ATP5PF*, *ATP5PO*, as well as the mitochondrially encoded genes *ATP6* and *ATP8*), which are all downregulated during treatment. Downregulation of these genes is consistent with the requirement of extensive oxidative phosphorylation during the peak of inflammation. Cells can increase oxidative phosphorylation by mitochondrial division or by increasing the density of cristae in mitochondria, which facilitates a higher density of oxidative phosphorylation complexes and enables mitochondria to be more efficient (102). Interestingly, enrichment of the pathways “SMAC XIAP regulated apoptotic response” and “rRNA modification in the mitochondrion,” which are connected *via* numerous protein-protein interactions, further suggests that the demand placed on the mitochondria has led to some mitochondrial dysfunction.

We used the pathways related to B cells and platelet activation to validate our approach. First, we demonstrated an increase in the expression of genes enriched in B cells, B cell activation and B cell receptor signaling during treatment (**Figures 4A, D**) and decrease in expression of genes enriched in platelet activation (**Figure 4B**). Second, we constructed induced regulatory pathways that incorporated TF with enriched TFBS. The regulatory interaction among PETGenes and their TFs demonstrates transcriptional regulation among PETGenes. High expression of genes enriched in “B cell activation”, at the end of treatment suggests up-regulation in B cells activation (**Figures S8** and **S9**) and low expression of genes enriched in “platelet activation” suggests down-regulation (**Figures S10**, **S11**).

Previous studies on TB treatment response (24, 25, 28–30) have only reported general changes in transcript levels in the peripheral blood, which are likely to be a collection of responses to drugs, reduction in bacterial load and inflammation. Four previous studies on human TB also reported a correlation between peripheral blood signatures and PET-CT [^18^F]FDG uptake measurements (28, 31, 33, 34). The uniqueness of our findings is that we directly correlated changes in transcripts levels in peripheral blood with [^18^F]FDG uptake in the lungs—a measure of inflammation—over time during TB treatment, taking into account the inherent intra- and inter-subject correlation structure of repeated measurements, thus our results describe more of the biology of lung inflammation in TB, as well as the temporal dynamics of transcriptional changes, and with statistical accuracy. Our models (linear mixed-effect models) identified subtle significant changes in transcript levels that are ignored in traditional gene expression analyses and provide robust results for downstream differential expression analysis and clustering.

### Limitations

Our study had some limitations. We used a linear-mixed effect model to analyze gene expression levels, which only considers linear correlation or relationship of gene expression with time. The linear-mixed effect model accounts only for the linear characteristics of measurements, ignoring other dynamics or patterns of gene expression and PET measurements which may be characteristic of TB. Additionally, transcriptomics only reveals changes in regulation of RNA levels which do not necessarily correspond to functional changes in proteins; nevertheless, it does provide insight into the regulatory responses, especially in longitudinal analyses. Lastly, the models correcting for cell proportions rely on computational deconvolution and not on differential counts, and the choice of which cell types to include in the models is difficult. Including too many variables (too many cell proportions) can weaken the inferential power of the models, and including cell types with little effect reduces power without any benefit. For future studies, we suggest using mixed-effect models with splines or other non-linear models with more time points. Another limitation is that PET-CT measures only glucose metabolism and not oxidative phosphorylation, nor does it provide a measure of bacterial burden; a direct measure of TB load would be ideal.

## Conclusion

In summary we have demonstrated that the resolution of inflammation in the lungs during TB treatment, measured with changes in PET-CT [^18^F]FDG uptake in the lungs, is positively correlated with down-regulation of genes enriched in “platelet activation”, “interferon and interleukin signaling”, and negatively correlated with up-regulation of genes enriched in “B cell activation” as well as many other pathways consistent with prior literature. These results validate our overall approach. In addition, we have shown that correcting for major cell type proportions using a leave-one-out approach allows identification of processes consistent with the remaining cell type. Lastly, we have identified “smooth muscle contraction” and pathways related to mitochondrial stress and dysfunction as highly enriched pathways. The extent of coordinated smooth muscle contraction gene expression suggests that the signal is derived from non-leukocyte origins, such as SPCs. The results obtained from our comprehensive pathway analyses provide important new insight into the pathobiology of TB. In future studies they could contribute to therapeutic target discovery and potential modulation of the host response to TB.

## Data Availability Statement

Publicly available datasets were analyzed in this study. This data can be found here: https://www.ncbi.nlm.nih.gov/geo/query/acc.cgi?acc=GSE89403.

## Ethics Statement

Approval was obtained from the Health Research Ethics Committee (HREC) of Stellenbosch University (registration number N10/01/013), to recruit patients and collect specimens. For the current study to re-analyze the PET-CT and mRNA expression data, we received a separate HREC approval (X18/09/029). The patients/participants provided their written informed consent to participate in this study.

## The Catalysis TB-Biomarker Consortium

**André G. Loxton,** Department of Science and Innovation/National Research Foundation Centre of Excellence for Biomedical Tuberculosis Research and South African Medical Research Council Centre for Tuberculosis Research, Division of Molecular Biology and Human Genetics, Faculty of Medicine and Health Sciences, Stellenbosch University, Cape Town, South Africa; **Annare Ellman,** Division of Nuclear Medicine, Department of Medical Imaging and Clinical Oncology, Faculty of Medicine and Health Sciences, Stellenbosch University, Cape Town, South Africa; **Bronwyn Smith,** Department of Science and Innovation/National Research Foundation Centre of Excellence for Biomedical Tuberculosis Research and South African Medical Research Council Centre for Tuberculosis Research, Division of Molecular Biology and Human Genetics, Faculty of Medicine and Health Sciences, Stellenbosch University, Cape Town, South Africa; **Caroline G. G. Beltran,** Department of Science and Innovation/National Research Foundation Centre of Excellence for Biomedical Tuberculosis Research and South African Medical Research Council Centre for Tuberculosis Research, Division of Molecular Biology and Human Genetics, Faculty of Medicine and Health Sciences, Stellenbosch University, Cape Town, South Africa; **Clifton E. Barry III,** Department of Science and Innovation/National Research Foundation Centre of Excellence for Biomedical Tuberculosis Research and South African Medical Research Council Centre for Tuberculosis Research, Division of Molecular Biology and Human Genetics, Faculty of Medicine and Health Sciences, Stellenbosch University, Cape Town, South Africa, Tuberculosis Research Section, Laboratory of Clinical Infectious Diseases, Division of Intramural Research, National Institute of Allergy and Infectious Diseases, National Institutes of Health, Bethesda, MD, United States, Wellcome Centre for Infectious Diseases Research in Africa, Institute of Infectious Disease and Molecular Medicine, Faculty of Health Science, University of Cape Town, Observatory 7925, South Africa; **David Alland,** Center for Emerging Pathogens, Department of Medicine, Rutgers-New Jersey Medical School, Rutgers Biomedical and Health Sciences, Newark, NJ, United States; **Friedrich Thienemann,** Wellcome Centre for Infectious Diseases Research in Africa, Institute of Infectious Disease and Molecular Medicine, Faculty of Health Science, University of Cape Town, Observatory 7925, South Africa, Department of Medicine, Groote Schuur Hospital, Faculty of Health Science, University of Cape Town, Cape Town, South Africa; **Gerard Tromp,** Department of Science and Innovation/National Research Foundation Centre of Excellence for Biomedical Tuberculosis Research and South African Medical Research Council Centre for Tuberculosis Research, Division of Molecular Biology and Human Genetics, Faculty of Medicine and Health Sciences, Stellenbosch University, Cape Town, South Africa; **Gerhard Walzl,** Department of Science and Innovation/National Research Foundation Centre of Excellence for Biomedical Tuberculosis Research and South African Medical Research Council Centre for Tuberculosis Research, Division of Molecular Biology and Human Genetics, Faculty of Medicine and Health Sciences, Stellenbosch University, Cape Town, South Africa; **James M. Warwick,** Division of Nuclear Medicine, Department of Medical Imaging and Clinical Oncology, Faculty of Medicine and Health Sciences, Stellenbosch University, Cape Town, South Africa; **Jill Winter,** Catalysis Foundation for Health, San Ramon, CA, United States; **Katharina Ronacher,** Department of Science and Innovation/National Research Foundation Centre of Excellence for Biomedical Tuberculosis Research and South African Medical Research Council Centre for Tuberculosis Research, Division of Molecular Biology and Human Genetics, Faculty of Medicine and Health Sciences, Stellenbosch University, Cape Town, South Africa; **Kim Stanley,** Department of Science and Innovation/National Research Foundation Centre of Excellence for Biomedical Tuberculosis Research and South African Medical Research Council Centre for Tuberculosis Research, Division of Molecular Biology and Human Genetics, Faculty of Medicine and Health Sciences, Stellenbosch University, Cape Town, South Africa; **Ilse Kant,** Division of Nuclear Medicine, Department of Medical Imaging and Clinical Oncology, Faculty of Medicine and Health Sciences, Stellenbosch University, Cape Town, South Africa; **Lani Thiart,** Department of Science and Innovation/National Research Foundation Centre of Excellence for Biomedical Tuberculosis Research and South African Medical Research Council Centre for Tuberculosis Research, Division of Molecular Biology and Human Genetics, Faculty of Medicine and Health Sciences, Stellenbosch University, Cape Town, South Africa; **Lance A. Lucas,** Department of Science and Innovation/National Research Foundation Centre of Excellence for Biomedical Tuberculosis Research and South African Medical Research Council Centre for Tuberculosis Research, Division of Molecular Biology and Human Genetics, Faculty of Medicine and Health Sciences, Stellenbosch University, Cape Town, South Africa; **Laura E. Via,** Tuberculosis Research Section, Laboratory of Clinical Infectious Diseases, Division of Intramural Research, National Institute of Allergy and Infectious Diseases, National Institutes of Health, Bethesda, MD, United States, Wellcome Centre for Infectious Diseases Research in Africa, Institute of Infectious Disease and Molecular Medicine, Faculty of Health Science, University of Cape Town, South Africa; **Lori E. Dodd,** Biostatistics Research Branch, National Institute of Allergy and Infectious Diseases, National Institutes of Health Bethesda, Maryland, United States; **Magdalena Kriel,** Department of Science and Innovation/National Research Foundation Centre of Excellence for Biomedical Tuberculosis Research and South African Medical Research Council Centre for Tuberculosis Research, Division of Molecular Biology and Human Genetics, Faculty of Medicine and Health Sciences, Stellenbosch University, Cape Town, South Africa; **Nelita Du Plessis,** Department of Science and Innovation/National Research Foundation Centre of Excellence for Biomedical Tuberculosis Research and South African Medical Research Council Centre for Tuberculosis Research, Division of Molecular Biology and Human Genetics, Faculty of Medicine and Health Sciences, Stellenbosch University, Cape Town, South Africa; **Patrick Dupont,** Laboratory for Cognitive Neurology, Department of Neurosciences, KU Leuven, Belgium, Division of Nuclear Medicine, Department of Medical Imaging and Clinical Oncology, Faculty of Medicine and Health Sciences, Stellenbosch University, Cape Town, South Africa; **Ray Y. Chen,** Tuberculosis Research Section, Laboratory of Clinical Infectious Diseases, Division of Intramural Research, National Institute of Allergy and Infectious Diseases, National Institutes of Health, Bethesda, MD, United States; **Robert J. Wilkinson,** Wellcome Centre for Infectious Diseases Research in Africa, Institute of Infectious Disease and Molecular Medicine, Faculty of Health Science, University of Cape Town, South Africa, Department of Medicine, Groote Schuur Hospital, Faculty of Health Science, University of Cape Town, Cape Town, South Africa, The Francis Crick Institute, London, United Kingdom, Department of Medicine, Imperial College London, United Kingdom; **Shubhada Shenai,** Center for Emerging Pathogens, Department of Medicine, Rutgers-New Jersey Medical School, Rutgers Biomedical and Health Sciences, Newark, NJ, United States; **Stephanie Griffith-Richards,** Division of Radiodiagnosis, Department of Medical Imaging and Clinical Oncology, Faculty of Medicine and Health Sciences, Stellenbosch University, Cape Town, South Africa; **Stephanus T. Malherbe,** Department of Science and Innovation/National Research Foundation Centre of Excellence for Biomedical Tuberculosis Research and South African Medical Research Council Centre for Tuberculosis Research, Division of Molecular Biology and Human Genetics, Faculty of Medicine and Health Sciences, Stellenbosch University, Cape Town, South Africa.

## Author Contributions

TO and GT carried out the computational analyses and drafted the manuscript. GW designed the original study, obtained funding for it and oversaw the study. GT designed and supervised the current bioinformatic analysis reported here. SMa interpreted PET-CT and provided quantitative data on PET-CT scans as well as clinical findings. DZ, ET, and FD contributed to the NGS analysis. SMe provided expertise and additional supervision on computational analyses. EM, NP, AL, LK, KR, and GW provided expert immunological and clinical knowledge on interpreting the results. HK and JW interpreted results and participated in drafting the manuscript. All authors contributed to the article and approved the submitted version.

## Funding

TO, SM, GW, and GT were supported by the South African Tuberculosis Bioinformatics Initiative (SATBBI), a Strategic Health Innovation Partnership grant from the South African Medical Research Council and South African Department of Science and Technology. SMa received funding from the EDCTP2 program supported by the European Union (grant number CDF1576). GW received funding from the South African National Research Foundation (SARChI TB Biomarkers #86535) and the South African Medical Research Council. AL is supported by the NRF-CSUR (Grant Number CSUR60502163639) and by the Centre for Tuberculosis Research from the South African Medical Research Council (SAMRC). HK was supported by the Faculty of Medicine and Health Sciences, Stellenbosch University, South Africa. The Catalysis Biomarker Consortium was funded by the Catalysis Foundation for Health, the Division of Intramural Research, National Institute of Allergy and Infectious Diseases, and the National Institute of Allergy and Infectious Diseases, International Collaborations in Infectious Disease Research.

## Conflict of Interest

The authors declare that the research was conducted in the absence of any commercial or financial relationships that could be construed as a potential conflict of interest.

## Supplementary Material

Supplementary Figure 1 Statistical modeling outline. **(A)** A total of 75 subjects diagnosed with active PTB, were followed up during treatment, and were considered cured at the end of treatment (EOT). [^18^F]FDG PET-CT scans were collected at Dx, week 4 (W04), and week 24 (W24). We observed a decrease in average [^18^F]FDG uptake levels, over time, during treatment. **(B)** We modelled [^18^F]FDG uptake levels, RNA-seq gene expression levels, and cell-type proportions, over time, using linear mixed-effects models. P, [^18^F]FDG uptake levels, as outcome; G, gene expression levels; T, time; C, cell-type proportions; and S, subjects. G, T and C were used as co-variables (see description of models in methods).

Supplementary Figure 2 Changes in MSLA levels, during PTB treatment. In this study MSLA levels were used as a PET metric for estimating [^18^F]FDG uptake in the lungs. PETC-CT data were available on 76 cured patients at diagnosis (Dx), week 4 (W04) and week 24 (W24) (see **Table 1** for more information). P-values were calculated using ANOVA. **(A)** Significant changes in the average MSLA levels of all cured subjects (n=75), during PTB treatment. **(B)** Temporal changes in MSLA levels during PTB treatment among cured subjects (n=75).

Supplementary Figure 3 Network illustrating gene-sharing relationship between Reactome pathways identified in the base model (no correction for cell proportion) using SetRank.

Supplementary Figure 4 Network of Reactome pathways identified by the “B cell” model (2.2). Description and symbols: see **Figure 3**.

Supplementary Figure 5 Network of Reactome pathways identified by the “CD8^+^ αβ T cell” model (2.3). Description and symbols: see **Figure 3**.

Supplementary Figure 6 Network of Reactome pathways identified by the “CD14^+^ monocyte” model (2.5). Description and symbols: see **Figure 3**.

Supplementary Figure 7 Network of Reactome pathways identified by the “neutrophil” model (2.5). Description and symbols: see **Figure 3**.

Supplementary Figure 8 Induced regulatory network of B cell genes, showing expression levels at Dx. Directed network showing genes as nodes, and interaction as edges. Blue broken lines, protein-protein interaction, thick brown lines, gene-protein synthesis interaction that is regulated by a transcription factor and thick green lines, regulatory interaction. Triangle, transcription factors; diamond, repressor; ellipse, transcribed gene; and octagon, interacting proteins. Red, high expression; dark red, very high expression; grey, moderate expression; and blue, low expression. PAX5 interacts with EBF1 to regulate the expression of target genes CD19, CD79A and BLK, which are involved in B cell development.

Supplementary Figure 9 Induced regulatory network of B cell genes, showing expression levels at W24. Directed network showing genes as nodes, and interaction as edges. Blue broken lines, protein-protein interaction, thick brown lines, gene-protein synthesis interaction that is regulated by a transcription factor and thick green lines, regulatory interaction. Triangle, transcription factors; diamond, repressor; ellipse, transcribed gene; and octagon, interacting proteins. Red, high expression; dark red, very high expression; grey, moderate expression; and blue, low expression. PAX5 interacts with EBF1 to regulate the expression of target genes CD19, CD79A and BLK, which are involved in B cell development. In the feedback loops, the transcription factors regulate target genes to synthesize proteins.

Supplementary Figure 10 Induced regulatory network of platelet genes, showing expression levels at Dx. Directed network showing genes as nodes, and interaction as edges. Lines: blue broken line, protein-protein interaction; thick brown line, gene-protein synthesis interaction that is regulated by a transcription factor; and thick green line, regulatory interaction. Symbols: transcription factors; diamond, repressor; ellipse, transcribed gene; and octagon, interacting proteins. Color: red, high expression; dark red, very high expression; grey, moderate expression; and blue, low expression.

Supplementary Figure 11 Induced regulatory network of platelet genes, showing expression levels at W24. Directed network showing genes as nodes, and interaction as edges. Lines: blue broken lines, protein-protein interaction; thick brown lines, gene-protein synthesis interaction that is regulated by a transcription factor; and thick green lines, regulatory interaction. Symbols: triangle, transcription factors; diamond, repressor; ellipse, transcribed gene; and octagon, interacting proteins. Color: red, high expression; dark red, very high expression; grey, moderate expression; and blue, low expression. In the feedback loops, the transcription factors regulate target genes to synthesize proteins.

Supplementary Table 1 List of genes with significant fit in Model 1. Genes with expression profiles correlated with Time. [Table_S01.xlsx]

Supplementary Table 2 List of GO biological processes significantly enriched in TimeGenes (Model 1). [Table_S02.xlsx]

Supplementary Table 3 List of genes with significant fit in Model 2.1. Genes with expression profiles correlated with [^18^F]FDG uptake. [Table_S03.xlsx]

Supplementary Table 4 List of genes in TimeGenes (Model 1), PETGenes (Model 2.1), and the intersection (overlap). [Table_S04.xlsx]

Supplementary Table 5 List of PETGenes with similar expression pattern over time, grouped into clusters. [Table_S05.xls]

Supplementary Table 6 Significance test for overlap between 320-probe treatment-specific signature from Bloom et al. (24) and PETGenes. [Table_S06.xlsx]

Supplementary Table 7 Significance test for overlap between 393-probe signature from Berry et al. (22) and PETGenes. [Table_S07.xlsx]

Supplementary Table 8 Significance test for overlap between “TB blood biomarkers in the lung” from Subbian et al. (85) and PETGenes. [Table_S08.xlsx]

Supplementary Table 9 Significance test for overlap between “list of significantly differentially expressed genes common to fibrotic nodules and cavitary lesions” from Subbian et al. (85) and PETGenes. [Table_S09.xlsx]

Supplementary Table 10 List of small signatures and overlap with 639 PETGenes.

Supplementary Table 11 List of transcription factor binding sites over-represented in PETGenes. [Table_S11.xlsx]

Supplementary Table 12 List of TFs, their target genes and TFBS identified. [Table_S12.xlsx]
