# Supplementary figures and images for: The Peripheral Blood Transcriptome Is Correlated With PET Measures of Lung Inflammation During Successful Tuberculosis Treatment
Source: Front Immunol. 2021 Feb 10;11:596173. doi: 10.3389/fimmu.2020.596173 (PMC7902901; doi:10.3389/fimmu.2020.596173)

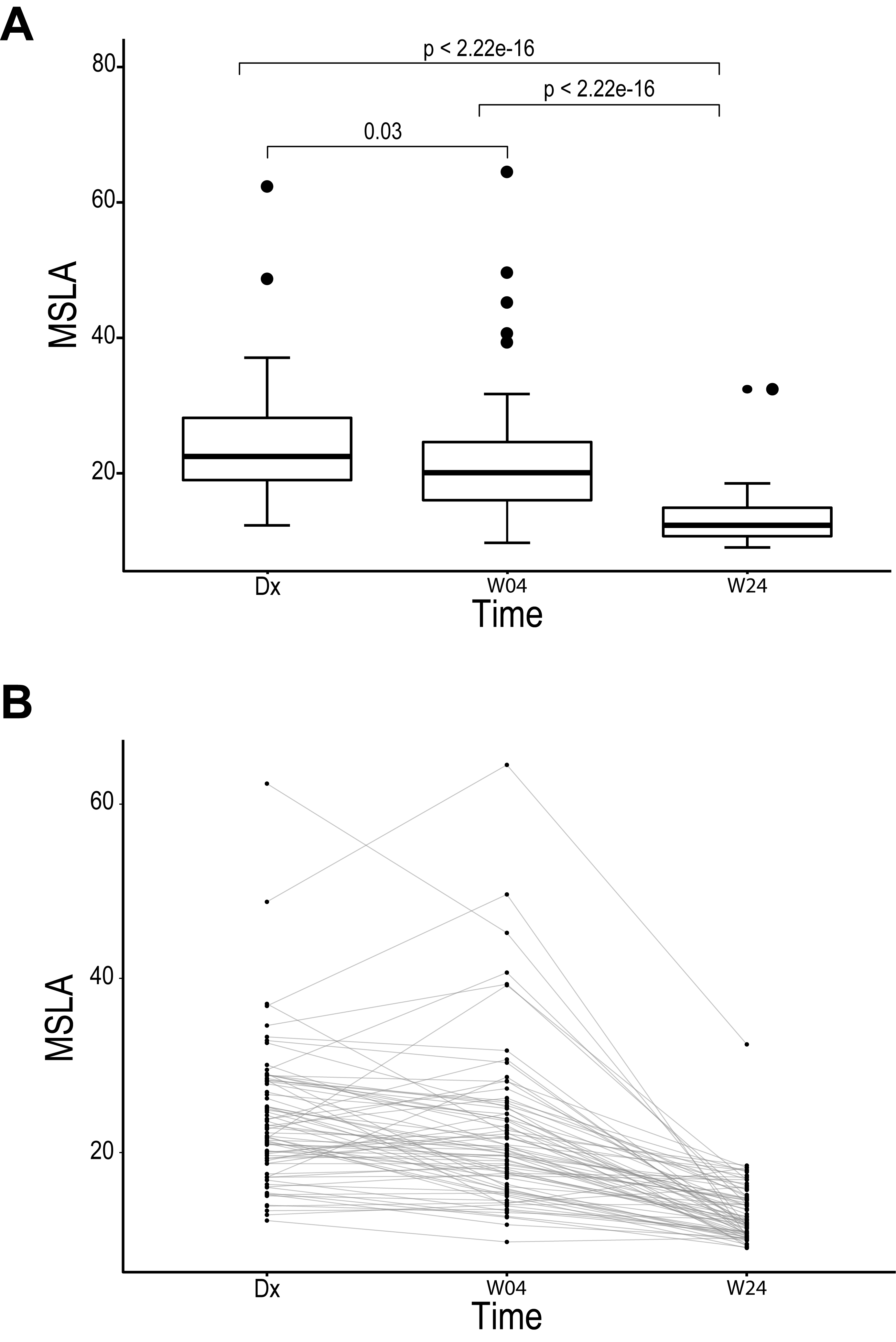

Supplement: Supplementary Figure 2 — Changes in MSLA levels, during PTB treatment. In this study MSLA levels were used as a PET metric for estimating [18F]FDG uptake in the lungs. PETC-CT data were available on 76 cured patients at diagnosis (Dx), week 4 (W04) and week 24 (W24) (see Table 1 for more information). P-values were calculated using ANOVA. (A) Significant changes in the average MSLA levels of all cured subjects (n=75), during PTB treatment. (B) Temporal changes in MSLA levels during PTB treatment among cured subjects (n=75). [file Image_2.tif]

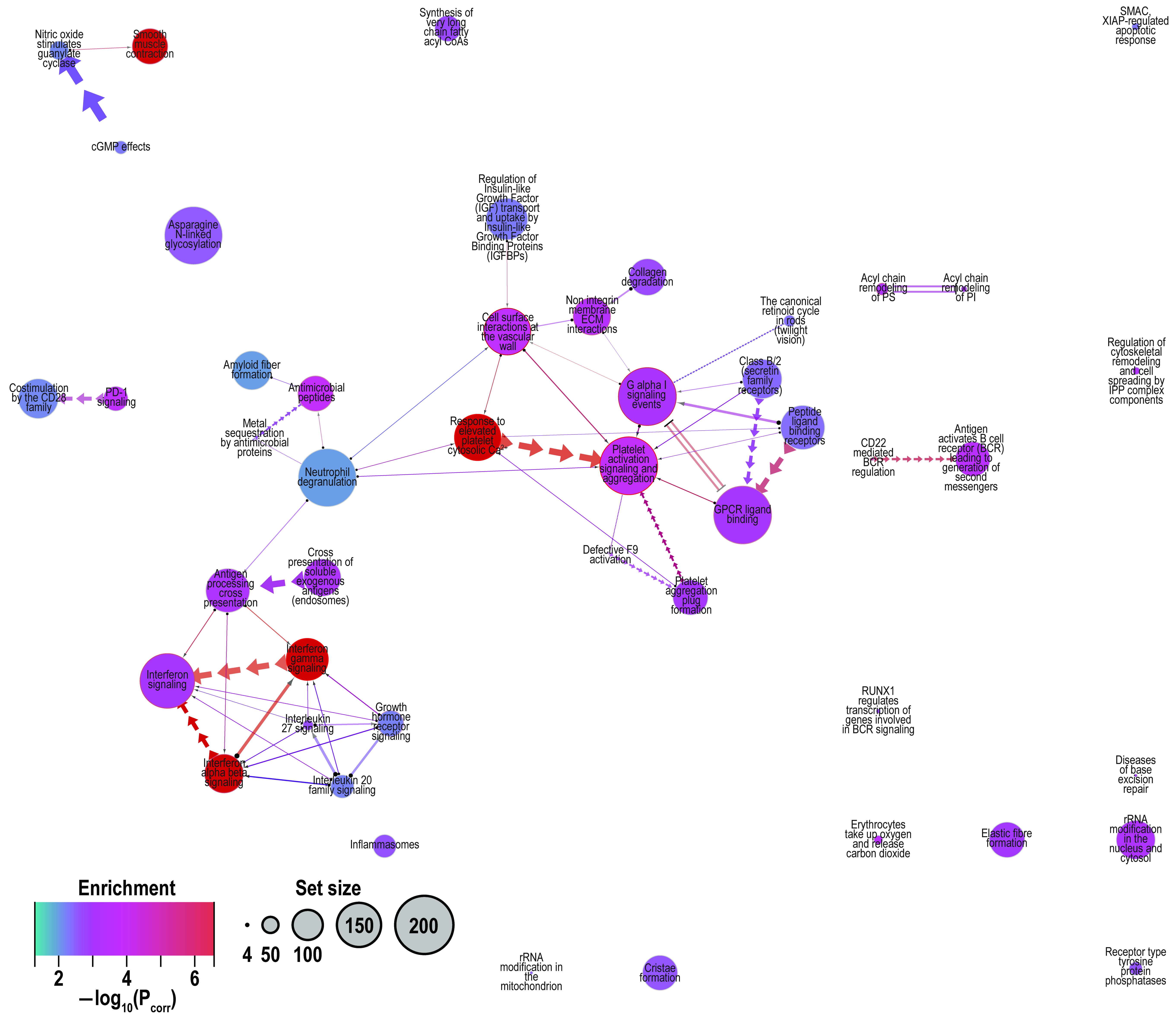

Supplement: Supplementary Figure 3 — Network illustrating gene-sharing relationship between Reactome pathways identified in the base model (no correction for cell proportion) using SetRank. [file Image_3.tif]

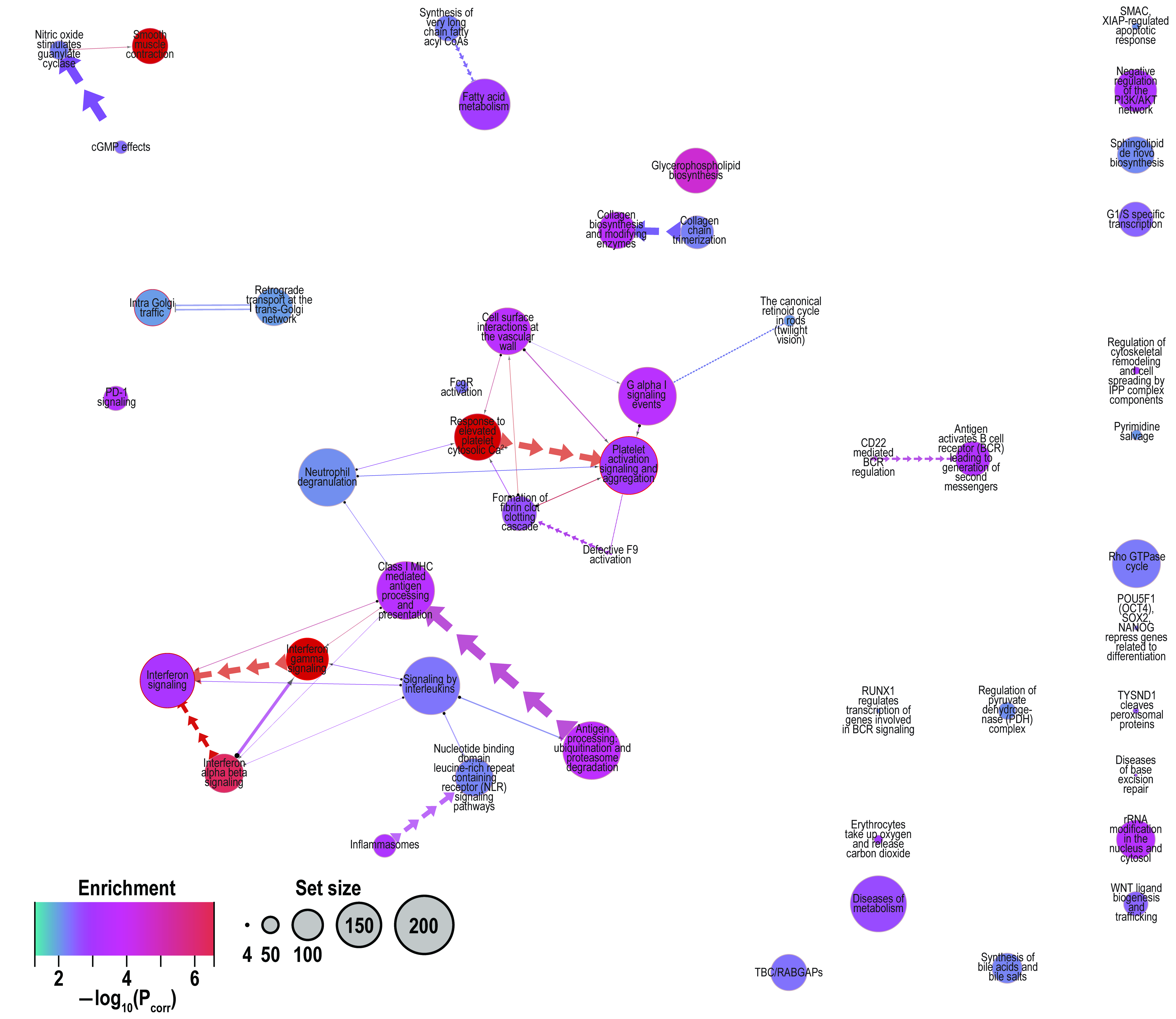

Supplement: Supplementary Figure 4 — Network of Reactome pathways identified by the “B cell” model (2.2). Description and symbols: see Figure 3 . [file Image_4.tif]

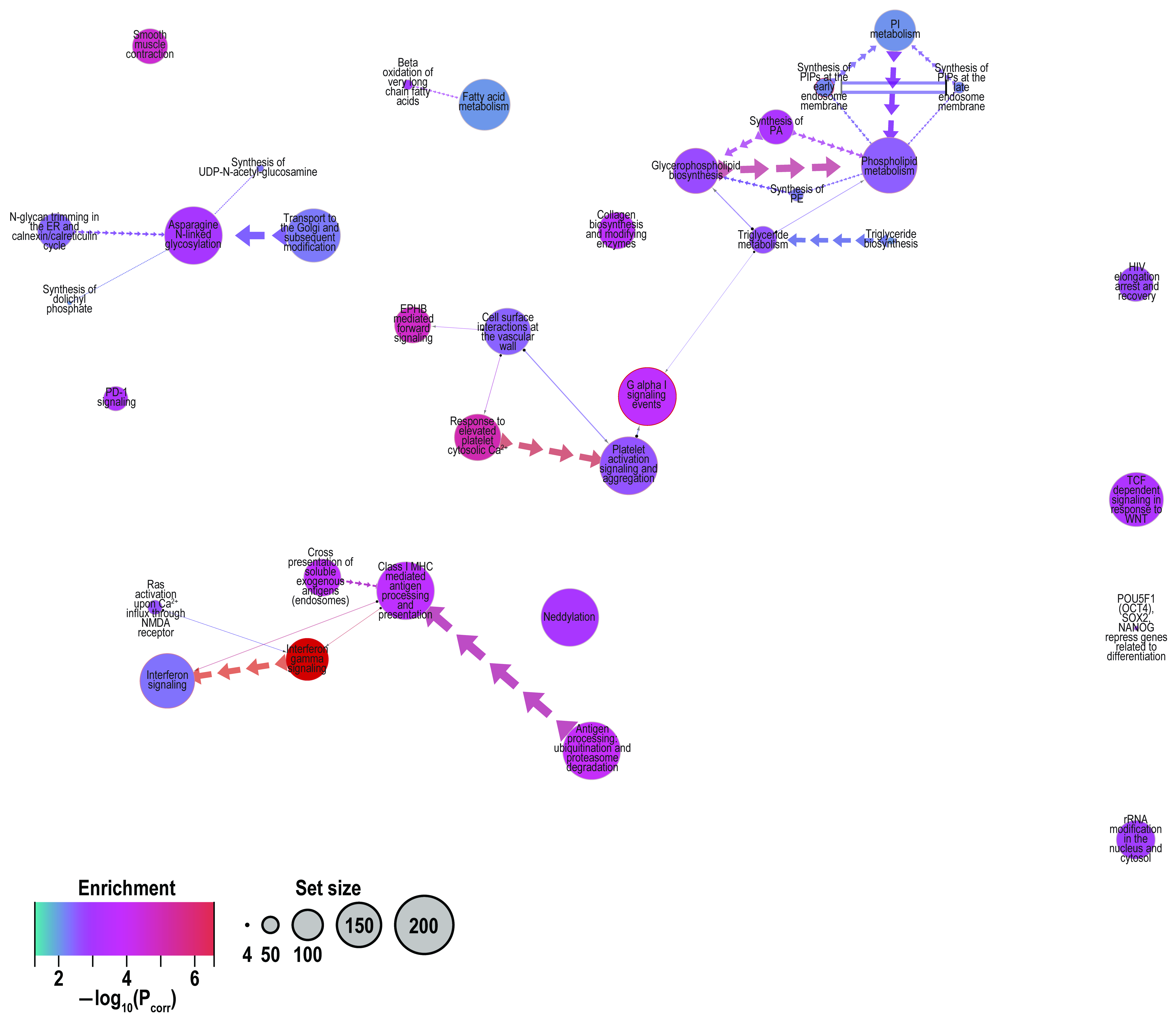

Supplement: Supplementary Figure 5 — Network of Reactome pathways identified by the “CD8+ αβ T cell” model (2.3). Description and symbols: see Figure 3 . [file Image_5.tif]

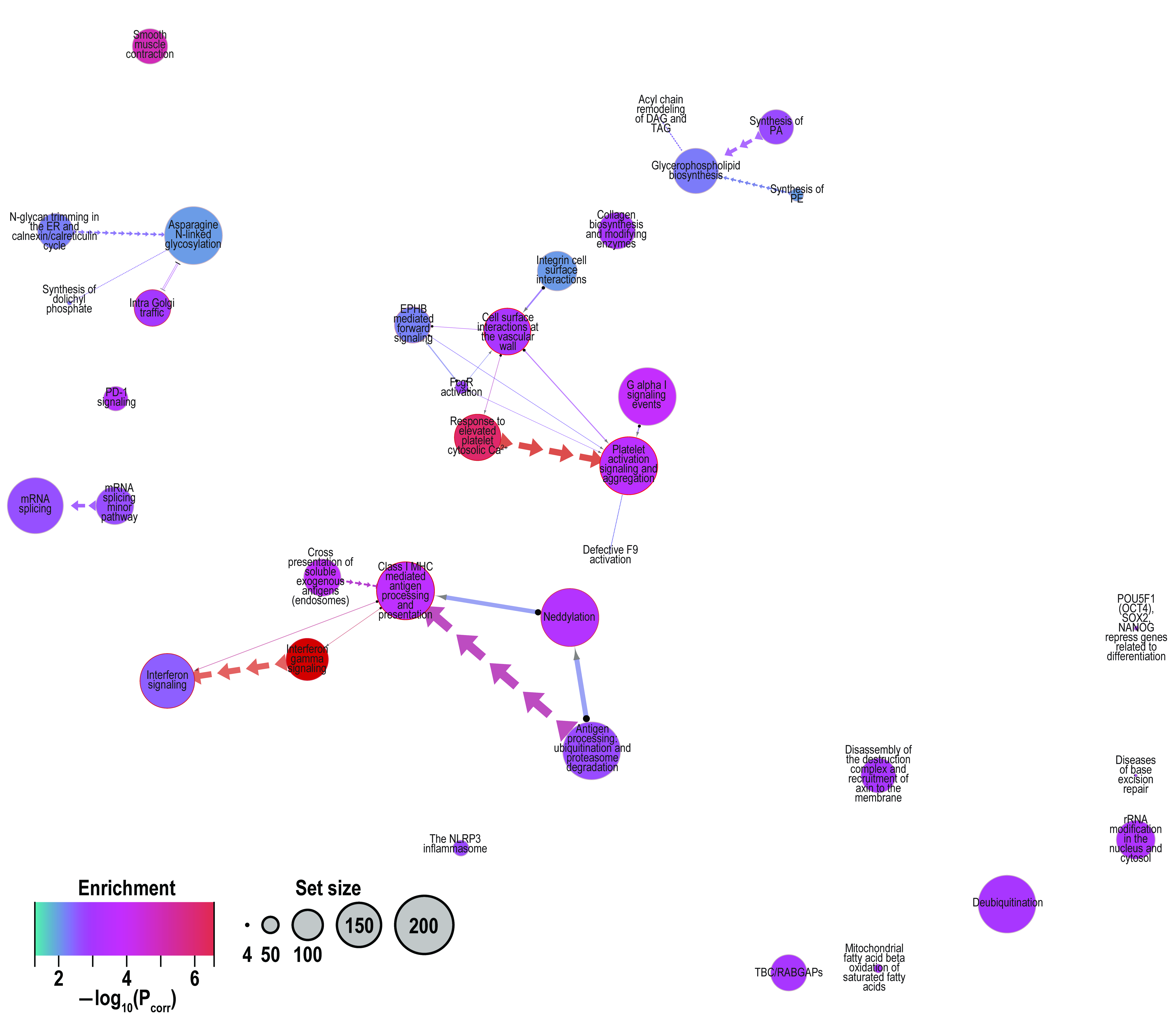

Supplement: Supplementary Figure 6 — Network of Reactome pathways identified by the “CD14+ monocyte” model (2.5). Description and symbols: see Figure 3 . [file Image_6.tif]

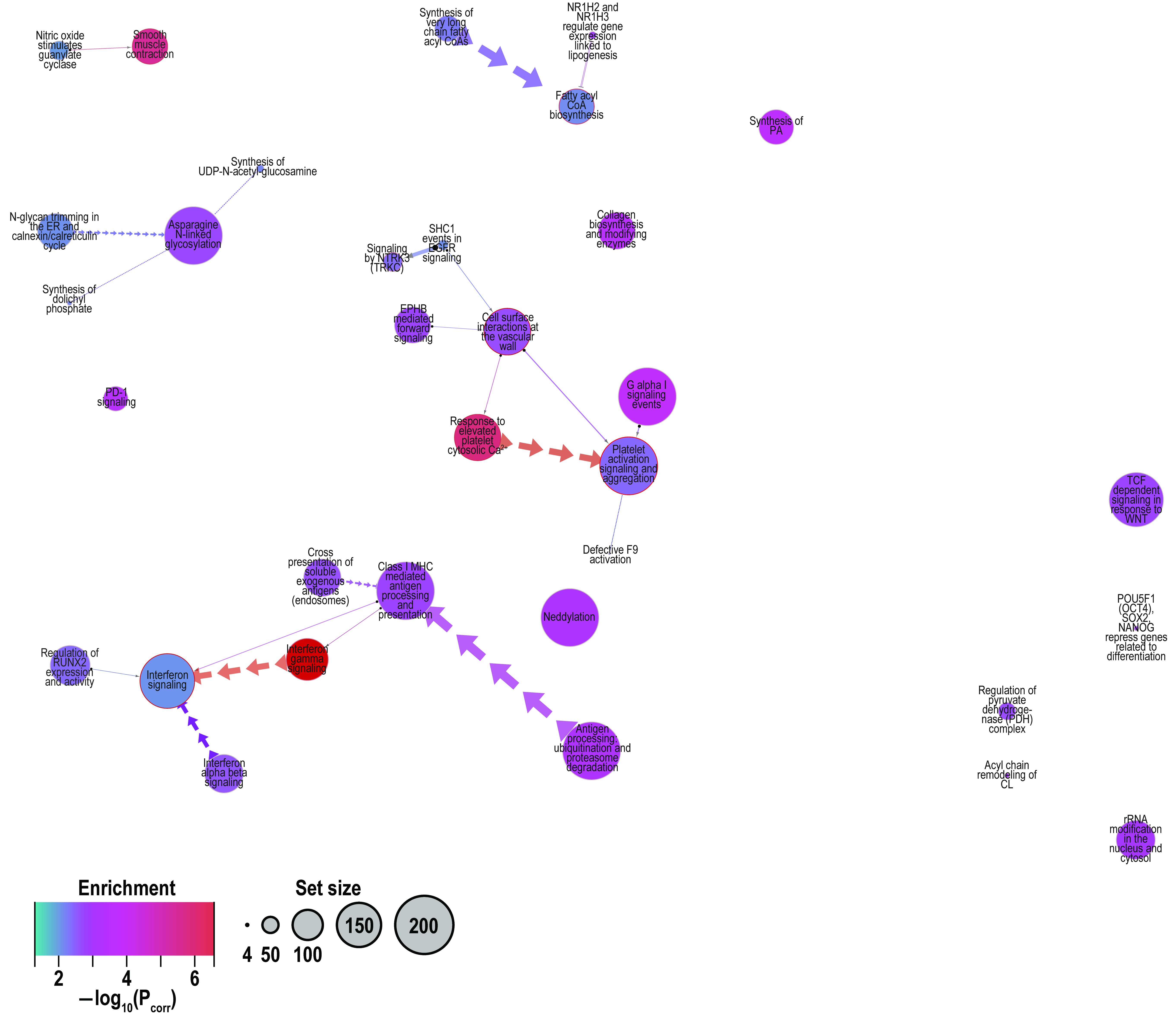

Supplement: Supplementary Figure 7 — Network of Reactome pathways identified by the “neutrophil” model (2.5). Description and symbols: see Figure 3 . [file Image_7.tif]

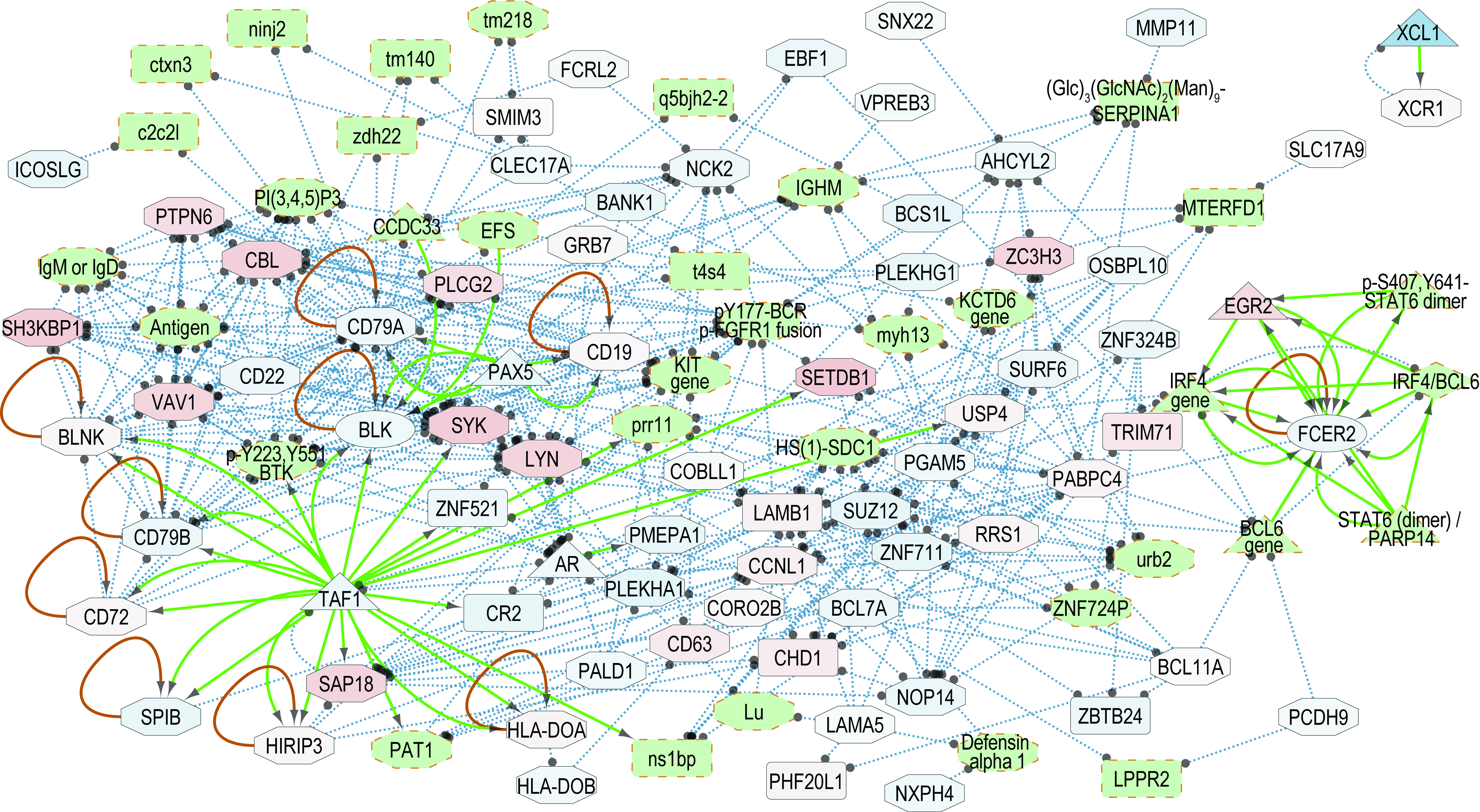

Supplement: Supplementary Figure 8 — Induced regulatory network of B cell genes, showing expression levels at Dx. Directed network showing genes as nodes, and interaction as edges. Blue broken lines, protein-protein interaction, thick brown lines, gene-protein synthesis interaction that is regulated by a transcription factor and thick green lines, regulatory interaction. Triangle, transcription factors; diamond, repressor; ellipse, transcribed gene; and octagon, interacting proteins. Red, high expression; dark red, very high expression; grey, moderate expression; and blue, low expression. PAX5 interacts with EBF1 to regulate the expression of target genes CD19, CD79A and BLK, which are involved in B cell development. [file Image_8.tif]

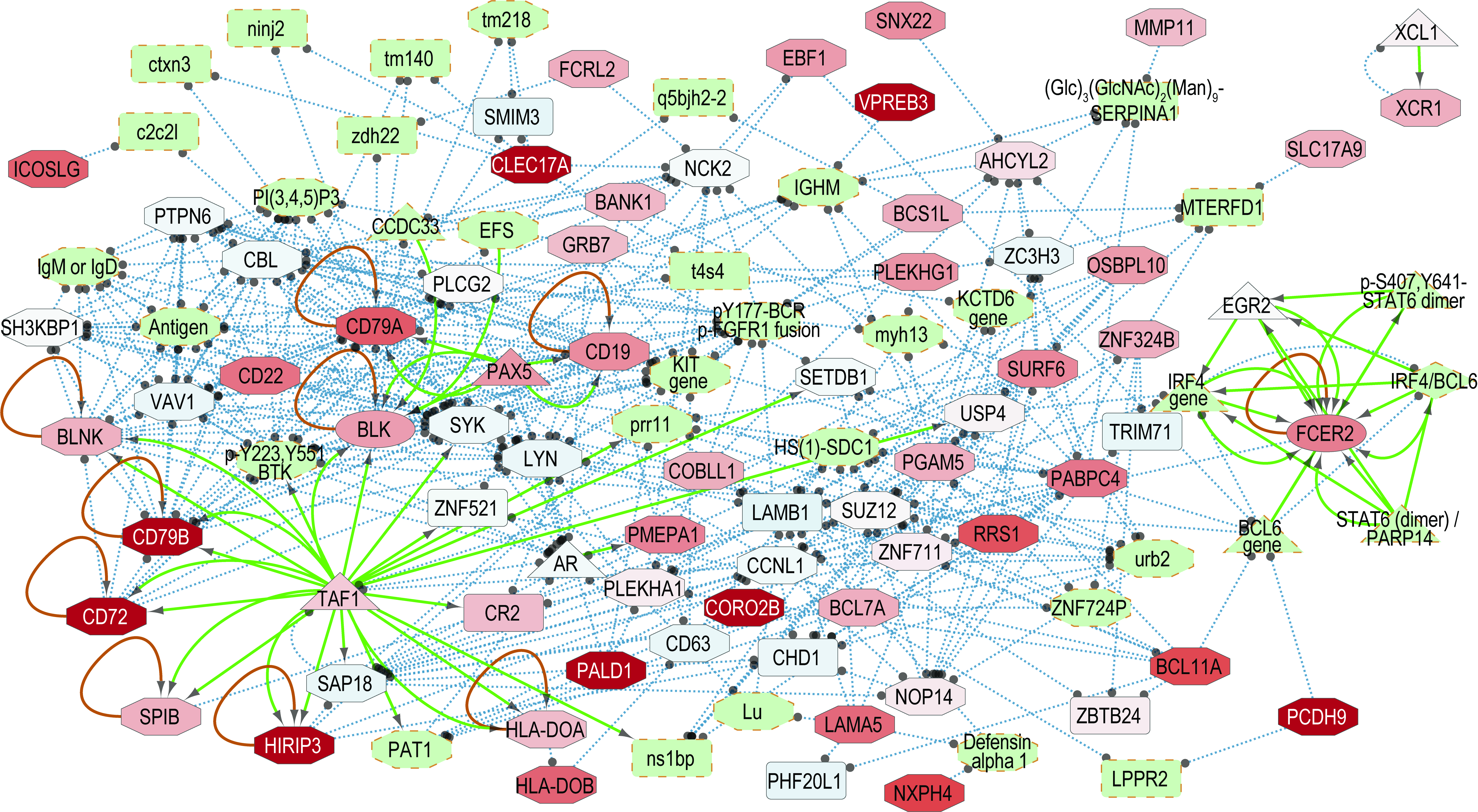

Supplement: Supplementary Figure 9 — Induced regulatory network of B cell genes, showing expression levels at W24. Directed network showing genes as nodes, and interaction as edges. Blue broken lines, protein-protein interaction, thick brown lines, gene-protein synthesis interaction that is regulated by a transcription factor and thick green lines, regulatory interaction. Triangle, transcription factors; diamond, repressor; ellipse, transcribed gene; and octagon, interacting proteins. Red, high expression; dark red, very high expression; grey, moderate expression; and blue, low expression. PAX5 interacts with EBF1 to regulate the expression of target genes CD19, CD79A and BLK, which are involved in B cell development. In the feedback loops, the transcription factors regulate target genes to synthesize proteins. [file Image_9.tif]

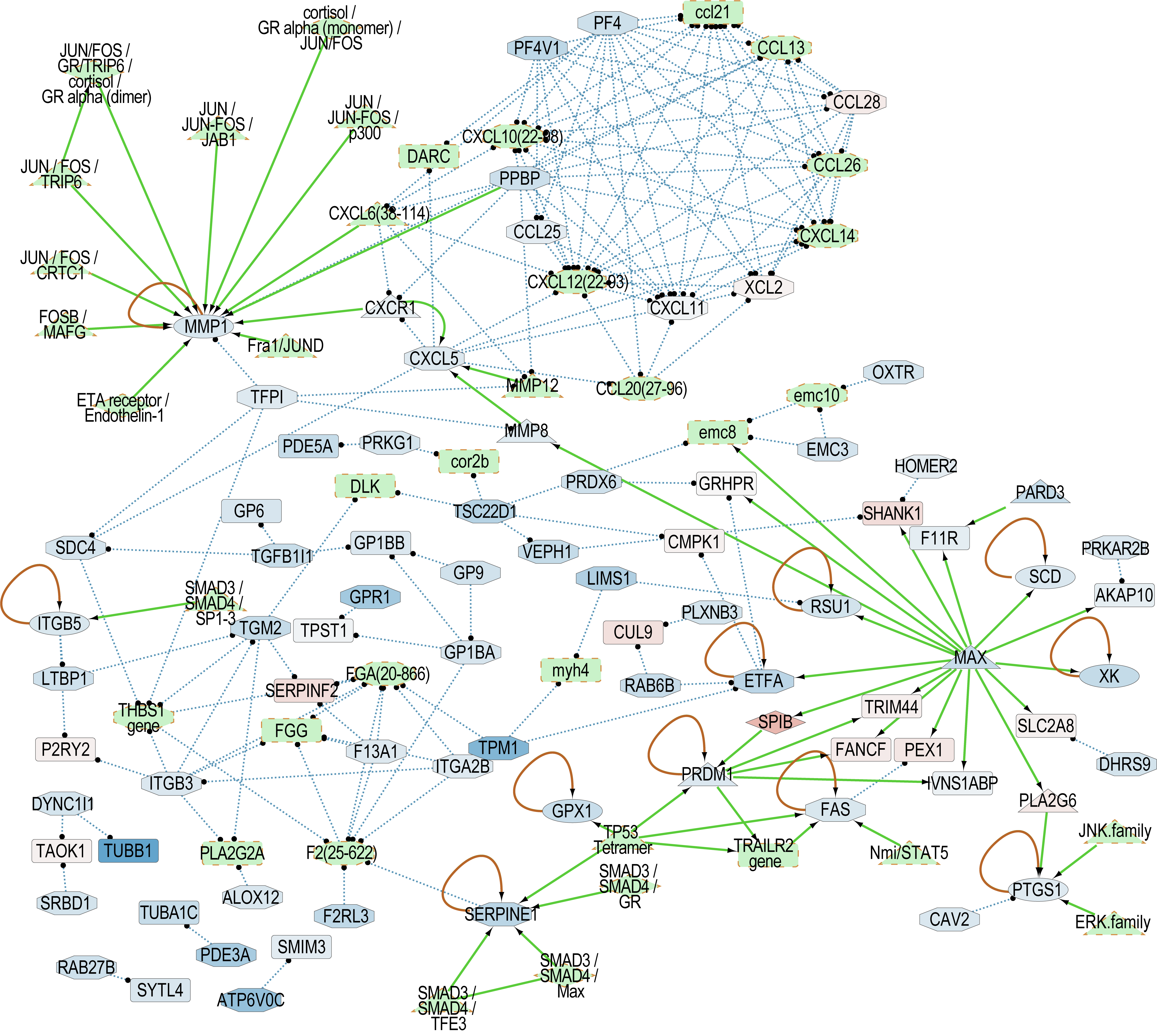

Supplement: Supplementary Figure 11 — Induced regulatory network of platelet genes, showing expression levels at W24. Directed network showing genes as nodes, and interaction as edges. Lines: blue broken lines, protein-protein interaction; thick brown lines, gene-protein synthesis interaction that is regulated by a transcription factor; and thick green lines, regulatory interaction. Symbols: triangle, transcription factors; diamond, repressor; ellipse, transcribed gene; and octagon, interacting proteins. Color: red, high expression; dark red, very high expression; grey, moderate expression; and blue, low expression. In the feedback loops, the transcription factors regulate target genes to synthesize proteins. [file Image_11.tif]
