# Supplementary material for: The Peripheral Blood Transcriptome Is Correlated With PET Measures of Lung Inflammation During Successful Tuberculosis Treatment
Source: Front Immunol. 2021 Feb 10;11:596173. doi: 10.3389/fimmu.2020.596173 (PMC7902901; doi:10.3389/fimmu.2020.596173)
Supplement: Supplementary file 24 [file DataSheet_1.pdf]

SUPPLEMENTARY METHODS

The Peripheral Blood Transcriptome is Correlated with PET Measures of Lung Inflammation during Successful Tuberculosis Treatment

T Odia, ST Malherbe, S Meier, E Maasdorp, L Kleynhans, N du Plessis, AG Loxton, DE Zak, E Thompson, FJ Duffy, H Kuivaniemi, K Ronacher, J Winter, G Walzl, G Tromp and the Catalysis TB-Biomarker Consortium

Participants in the Original Catalysis TB Biomarker Study

Pulmonary TB patients for the original study were recruited from the local communities or at Tygerberg Academic Hospital in Cape Town, South Africa. We followed strictly the inclusion and exclusion criteria defined in the original study protocol.

The exclusion criteria for study participants were:

- Living in the study area for less than 3 months
- No permanent address.
- Individuals with an hemoglobin < 10 g/l
- Individuals participating in drug or vaccine trial
- The presence of known other major medical conditions including Diabetes Mellitus or raised random blood glucose levels (a random blood glucose level was determined prior to the PET scan), chronic bronchitis/emphysema/asthma requiring steroid therapy, cancer, pregnancy (current or within 3 months), steroid therapy within past 6 months, known alcohol abuse (>3 alcoholic beverages/day, where one beverage includes one bottle of beer, one glass of wine, one tot of spirits) or any known illicit drug use
- Individual started treatment for active TB prior to entering this study

The inclusion criteria were:

- Member of local community where Stellenbosch University has permission to recruit TB patients at local health care clinics, or an attendee of the Infectious Diseases Clinics at Tygerberg Academic Hospital.
- Willingness to give consent
- Willingness to submit to HIV status testing or having their HIV status disclosed to the study field workers
- HIV-negative
- Age between 16 and 70 years
- Either 1) newly diagnosed pulmonary TB; or 2) recurrent TB with any previous TB treatment completed at least 12 months prior to recruitment. Where TB disease status was confirmed by two separate positive sputum smears and PCR for DNA of bacteria in the Mtb complex.

The study patients received treatment as prescribed by the South African National Tuberculosis Programme, based on WHO guidelines. This consisted of 6 months combination therapy: two months intensive phase of rifampicin, isoniazid, pyrazinamide and ethambutol daily, followed by four months continuation phase of rifampicin and isoniazid daily under direct observation. The dosages of these medications were adjusted based on the weight of the patient as shown in the Table below:

Dosages used in standard TB treatment

Drug	Dosage (mg/day) patient < 50kg	Dosage (mg/day) patient > 50kg
Isoniazid	320	400
Rifampicin	480	600
Pyrazinamide	1,000	1,250
Ethambutol	800	1,200

Of the 75 cured subjects, 56 (75%) received the standard TB treatment. One subject was treated for 1 month with rifampicin, isoniazid, pyrazinamide, ethambutol, and streptomycin, followed by the standard treatment (standard + streptomycin). Four subjects were given extended treatment consisting of 2 months with rifampicin, isoniazid, pyrazinamide and ethambutol followed by 6 months with rifampicin and isoniazid (extended 8). Twelve subjects were treated for 1 month with rifampicin, isoniazid, pyrazinamide, ethambutol, and streptomycin, followed by 3 months with rifampicin, isoniazid, pyrazinamide and ethambutol, and then 5 months with rifampicin and isoniazid (extended 8 + streptomycin). Another subject was placed on rifampicin, isoniazid, pyrazinamide and ethambutol for 12 months (extended 12). One subject was treated with a multiple drug resistance regimen, i.e., treated for 24 months and received second-line drugs (MDR).

The treatment is summarized in the Table below:

Summary of treatment regimens

Description	Rifampicin	INH	PZA	Eth	Streptomycin	Second line	Duration (months)	Count (n)
Standard	Y	Y	Y	Y			6	56
Standard + Streptomycin	Y	Y	Y	Y	Y		6	1
Extended 8	Y	Y	Y	Y			8	4
Extended 8 + Streptomycin	Y	Y	Y	Y	Y		8	12
Extended 12	Y	Y	Y	Y			12	1
MDR						Y	20	1
